# Developmental trajectory of social influence integration into perceptual decisions in children

**DOI:** 10.1073/pnas.1808153116

**Published:** 2019-01-28

**Authors:** Imogen Large, Elizabeth Pellicano, Andreas Mojzisch, Kristine Krug

**Affiliations:** ^a^Department of Physiology, Anatomy and Genetics, University of Oxford, Oxford OX1 3PT, United Kingdom;; ^b^Department of Educational Studies, Macquarie University, North Ryde, NSW 2109, Australia;; ^c^Centre for Research in Autism and Education, UCL Institute of Education, University College London, London WC1H 0AL, United Kingdom;; ^d^Institute of Psychology, University of Hildesheim, D-31141 Hildesheim, Germany

**Keywords:** development, autism, decision making, drift diffusion, social bias

## Abstract

The opinions of others have a profound influence on decision making in adults. The impact of social influence appears to change during childhood, but the underlying mechanisms and their development remain unclear. We tested 125 neurotypical children between the ages of 6 and 14 years on a perceptual decision task about 3D-motion figures under informational social influence. In these children, a systematic bias in favor of the response of another person emerged at around 12 years of age, regardless of whether the other person was an age-matched peer or an adult. Drift diffusion modeling indicated that this social influence effect in neurotypical children was due to changes in the integration of sensory information, rather than solely a change in decision behavior. When we tested a smaller cohort of 30 age- and IQ-matched autistic children on the same task, we found some early decision bias to social influence, but no evidence for the development of systematic integration of social influence into sensory processing for any age group. Our results suggest that by the early teens, typical neurodevelopment allows social influence to systematically bias perceptual processes in a visual task previously linked to the dorsal visual stream. That the same bias did not appear to emerge in autistic adolescents in this study may explain some of their difficulties in social interactions.

## Significance

Social influence biases even simple perceptual decisions across a range of contexts. The development and mechanism of such biases remain unclear. We systematically examined the developmental course of social influence bias exerted by another person on perceptual decisions in children between 6 and 14 years old. To probe underlying mechanisms, we applied the computational model drift diffusion to behavioral response data. Our results show that although young neurotypical children seem to be unaffected by social influence, adolescents develop a systematic bias of their responses in the direction of social influence and appear to do so by integrating social information into visual perceptual processing. The same pattern of results did not emerge in age-matched autistic children, suggesting a different developmental trajectory.

Decisions in everyday life rarely occur in a social vacuum. The scientific exploration of social influence on judgment and decision making rose to prominence with Solomon Asch’s seminal experiments in the 1950s (1, 2). He demonstrated that when making a judgment about the appearance of a visual stimulus, adults would at times conform to a majority opinion, even when it was blatantly wrong. Surprisingly, when asked why they went along with the incorrect group decision, some participants insisted that they had seen the visual stimulus as they reported. Altered visual processing could be one mechanism by which information obtained from others in a social context affects the decision process. An alternative mechanism would be conformity to the opinions of others operating solely at the decision level. Several studies have supported a model of social influence in which changes in sensory processing and perception contribute to biases of perceptual decisions under social influence—at least in adults (3–7). Here, we employ a widely used perceptual decision task involving the discrimination of 3D-motion figures and examine this model in children who follow typical and atypical developmental trajectories.

Although it is well known that adolescents are generally more susceptible to social influence than other age groups (8), social influence appears to exert an effect from an early age (9, 10). Berenda (11) provided some of the first evidence for behavioral conformity to incorrect group judgments in 7- to 10-y-old children. Children below the age of 7 y (and as young as 3 y) are able to make judgments about socially delivered information, preferring the more accurate and reliable advisor (12) and modify their judgments accordingly (13). However, there are also developmental differences in conforming to a group consensus: whereas 7-y-olds already use social information in both public and private perceptual decisions, they do so to a lesser extent than 10- and 13-y-olds or adults (14). Furthermore, the type of advisor can affect the degree of conformity such that teenagers are more influenced by their peers than by adults when assessing risky decisions (15), but it is unclear how this advisor-related pattern of social influence changes more generally across development. This body of work indicates that even though young children can make judgments about social influence, susceptibility to social conformity may change during development and in different social and task contexts.

Insights regarding the nature and extent of susceptibility to social influence can also be gleaned from individuals who have marked difficulties in interacting and communicating with others. Autistic children can show difficulties orienting toward other people from early childhood (16): in initiating and responding to bids for joint shared attention (e.g., ref. 17) and in making and keeping friends (18). [Because identity-first language rather than person-first language is the preferred term of many people on the autism spectrum (19) and their parents (20), we use this terminology in this paper.] These social difficulties suggest that autistic children and adults may be less influenced by the opinions of others. In a recent study using a modified “Asch experiment” for school-age children, with a single adult reporting what “most people” think, there was strong evidence of conformity in the neurotypical group, but the majority of autistic children did not conform to incorrect advice, instead choosing the correct perceptual response (21). These results are in line with current theoretical accounts, which propose that autistic children rely more on incoming sensory evidence and are less susceptible to top-down (including social) influences (22–24).

**Fig. 1.**
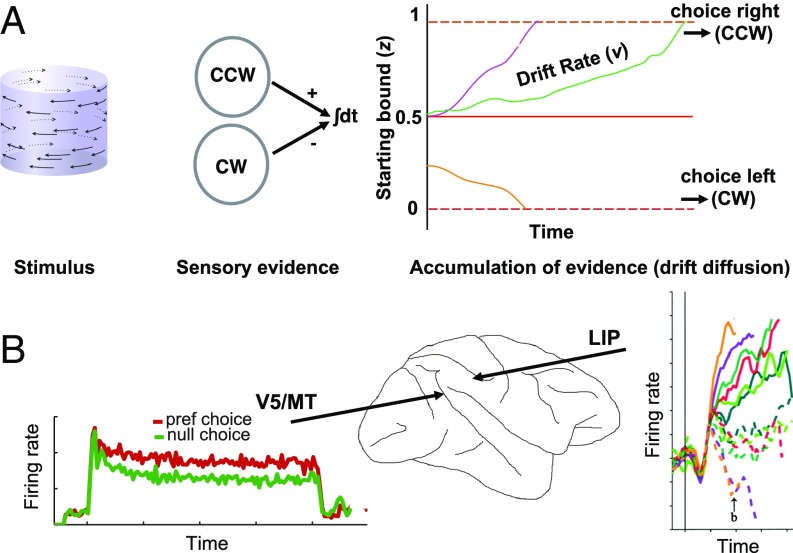
Drift diffusion model for perceptual decision making. (*A*) Schematic representation of the drift diffusion model. While viewing a visual stimulus (here, a rotating SFM cylinder), incoming sensory evidence is processed in pools of visual neurons specific to the perceptual appearance of rotation direction [e.g., clockwise (CW) or front surface moving left; counterclockwise (CCW) or front surface moving right]. When making a choice between two potential perceptual interpretations (e.g., CCW or CW), circuits in the brain are thought to accumulate supporting and opposing sensory evidence over time in the form of a decision variable. In the model, based on the theory of Brownian motion representing patterns of neuronal activity (98), the decision variable drifts in a Brownian fashion until it reaches a threshold (decision bound), resulting in a decision (e.g., participant reports CCW by indicating right). The drift rate, *v,* describes the rate of evidence accumulation and is affected by the available sensory evidence. If the starting point, *z*, for a decision differs significantly from the midpoint between the two potential choices, this suggests an initial decision bias. Drift rate and starting point can be predicted from the RT distribution in perceptual decision tasks. Orange, green, and pink lines illustrate schematically potential evidence accumulation paths. (*B*) Parameters *v* and z of the drift diffusion model might be linked to specific neuronal processes in the primate brain (30). When rhesus macaques make decisions about visual motion stimuli of different signal strength, neurons in the lateral intraparietal (LIP) area show firing rates that rise during the trial and might accumulate perceptual evidence [*Right* image reproduced with permission from ref. 31.]. Changes in starting point, *z*, were linked to this sensorimotor level of brain processing. In contrast, visual cortical areas represent sensory evidence that might feed into the accumulation process (99). Changes at this level of processing should affect the drift rate, *v*. For the 3D stereomotion task in this study, representations of perceptual evidence in visual area V5/MT show steady firing rates during stimulus presentation [*Left* (100)].

Computational models can link behavioral data to specific levels of brain processing. Drift diffusion models allow us to make such inferences about brain mechanisms that underpin perceptual decisions by using behavioral data (25–29). This model formally distinguishes changes in the rate at which sensory evidence accumulates (drift rate), indicating a bias in sensory processing, and biases that affect the start position of the accumulation process (starting point), which are thought to affect decision, but not sensory, processing (Fig. 1). Drift diffusion models have implicated altered sensory processing as one source of bias in decision making under social influence in adults by showing changes in the drift rate with social influence (6, 7). Drift diffusion models have been linked to specific neural processes and circuits in the cerebral cortex of primates (30). Neuronal signals in sensorimotor areas represent the integration of evidence in a decision variable for motion discrimination (31, 32), while neuronal signals in occipital areas represent visual perceptual evidence (33). With regard to the visual task in the present study, extrastriate visual area V5/MT in macaques contributes directly to the perception of such stereomotion figures (34, 35); macaque V5/MT is thought to be homologous to human hMT+ (36, 37).

The current study systematically investigated the development and mechanisms of social influence biases in neurotypical children and autistic children between the ages of 6 and 14 y. Children judged the direction of rotation of stereomotion-defined cylinders—a visual task in which children were competent, performing like adults, and for which the neural substrate has been well defined in primates. We used a single advisor (peer or adult) to investigate the perceptual and decision bias effects of social influence, including the impact of the advisor’s relative age, on decision making. This paradigm minimized effects of group pressure, focusing instead on informational effects of social influence (38). Fitting behavioral responses with a psychometric function, we quantified the bias due to social influence. To make inferences about the level of processing at which social influence is integrated into the decision process, we fit a drift diffusion model to reaction time (RT) data.

## Results

### Stereo Thresholds of Children Between 6 and 14 y of Age Are Adult-Like.

**Fig. 2.**
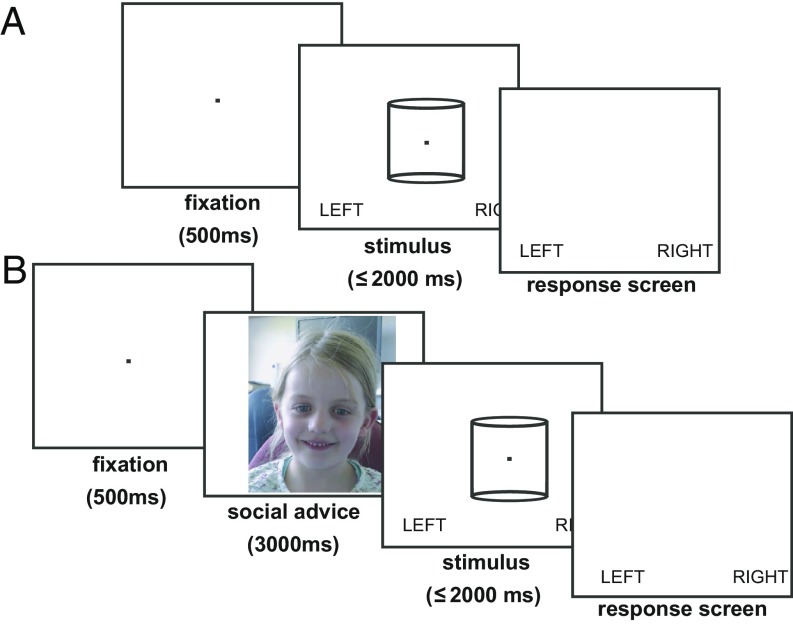
Trial structure of the RT tasks. (*A*) Visual task for measuring stereomotion thresholds with the rotating SFM cylinder stimulus. A fixation point was presented for 500 ms, followed by the SFM stimulus for up to 2,000 ms. The SFM cylinder was shown with one of seven cylinder disparities (typically from +0.03° to −0.03° in steps of 0.01°) pseudorandomly interleaved. Participants were asked to indicate the direction of cylinder rotation (direction of dot motion on front surface) by pressing the touch screen in the corresponding place (left or right) as quickly and as accurately as possible after stimulus onset. (*B*) Visual task with social influence. A fixation point was presented for 500 ms, followed by a 3,000-ms video of a gender-matched advisor stating that the forthcoming stimulus would either be spinning left or right. This was followed by a rotating SFM stimulus for up to 2,000 ms. Again, the cylinder was presented with one of seven different levels of cylinder disparity pseudorandomly interleaved. Participants were asked to indicate the direction of cylinder rotation by pressing the touch screen in the corresponding place as quickly and as accurately as possible (photo of child is from the advice videos, used with parental permission).

**Fig. 3.**
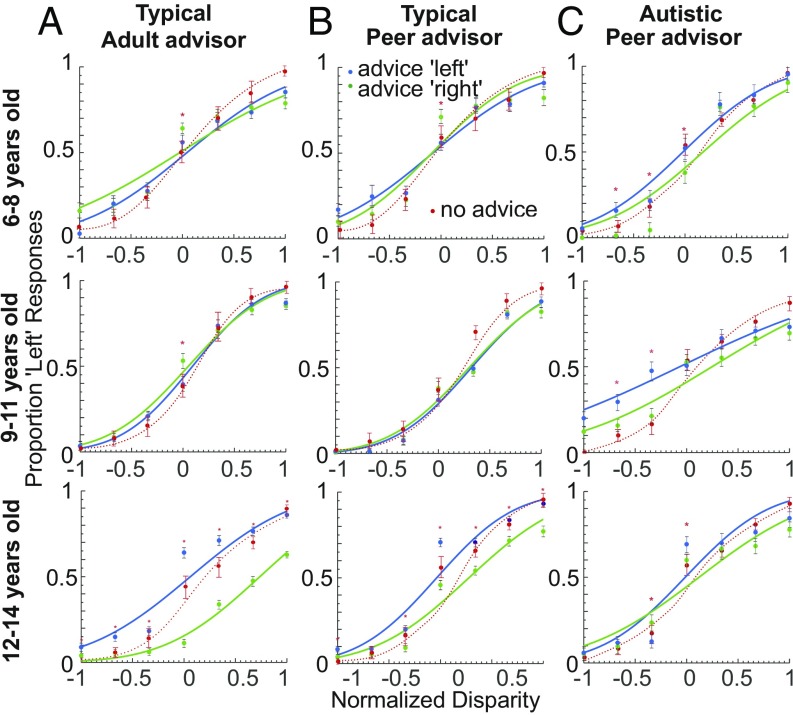
Effect of social influence on perceptual decisions. (*A*) Perceptual decisions of neurotypical children with an adult as advisor. Children made judgments about the rotational direction of an SFM cylinder after receiving advice from a gender-matched adult. We normalized the individual child’s range of cylinder disparities and then averaged behavioral responses for each level of disparity signal, for each age group. Psychometric responses were fitted with two cumulative Gaussian functions, separately for advice left and advice right. A systematic, consistent bias in the direction of social influence was apparent for the 12- to 14-y-old neurotypical children. In red, results are shown for the visual task without social influence. Error bars depict SEM. An asterisk indicates a significant difference between responses for leftward vs. rightward social influence at a given disparity (Wilcoxon rank sum, *P* < 0.0001, Bonferroni corrected for significance at *P* < 0.05). (*B*) Perceptual decisions for neurotypical children with an age- and gender-matched peer advisor. Results are comparable to those in *A* for an adult advisor. (*C*) Perceptual decisions for autistic children with an age- and gender-matched peer advisor. Children were matched in IQ to the neurotypical group in *B*. There is a weak bias with social influence for younger autistic children, but no bias for 12- to 14-y-olds. Conventions as in *A*.

For 103 neurotypical children across three age groups (6 to 8, 9 to 11, and 12 to 14 y) from our total sample of 125 neurotypical children, we separately assessed 3D stereomotion discrimination threshold. Participants were asked to report the direction of rotation of a structure-from-motion (SFM) cylinder via a touch screen as quickly and as accurately as possible (visual task; Fig. 2*A*). The binocular disparity assigned to the SFM cylinder defined the direction of rotation and rendered it either more, or less, ambiguous on different trials. On each trial, we recorded the behavioral response about the cylinder’s rotation direction and the RT. Psychometric functions were fitted for each participant. We also tested 30 autistic children on the same visual task, who were age and IQ matched to a subgroup of the neurotypical children (“peer-advice group” for subsequent social influence experiment). When visual stimuli were presented without social influence, children’s binocular disparity thresholds were comparable to those reported previously in adults (around 0.02°, refs. 39–41). There were no significant differences in stereo thresholds with age, diagnosis, or assigned experimental group (Table 1) (see also red curve for psychometric functions in Fig. 3). Thus, children across all age groups and regardless of an autism diagnosis could carry out the basic visual stereomotion task upon which our social experiment builds. There were no group differences in visual task performance that might subsequently have influenced the interaction between visual task and social influence.

**Table 1.** Psychometric thresholds for stereo acuity by age and experimental group

Age (y)	Neurotypical (combined) median ± SD	Neurotypical (peer-advice group) median ± SD	Neurotypical (adult advice group) median ± SD	Autistic median ± SD
6–8	0.009° ± 0.014 (*n* = 38)	0.009° ± 0.008 (*n* = 18)	0.009° ± 0.017 (*n* = 20)	0.008° ± 0.014 (*n* = 9)
9–11	0.011° ± 0.012 (*n* = 27)	0.019° ± 0.009 (*n* = 9)	0.008° ± 0.014 (*n* = 18)	0.014° ± 0.013 (*n* = 11)
12–14	0.010° ± 0.014 (*n* = 38)	0.017° ± 0.009 (*n* = 12)	0.007° ± 0.015 (*n* = 26)	0.021° ± 0.010 (*n* = 10)

Participants discriminated the direction of rotation of an SFM cylinder stimulus (visual task; Fig. 2*A*). The SD of the Gaussian curve fitted to behavioral responses provided the threshold binocular disparity (in degrees) (see ref. 101). Neurotypical participants (column 2) were also analyzed separately according to subsequent experimental groups in the social influence study (peer-advice and adult-advice groups) (columns 3 and 4). There were no differences in stereo thresholds with age, diagnosis, or experimental group (peer or adult advice) [ANOVA; age *F*(2,124) = 0.48, *P* = 0.620; diagnosis *F*(1,124) = 0.04, *P* = 0.838; experimental group *F*(1,124) = 0.39, *P* = 0.533] and no significant interactions. A direct comparison between the matched neurotypical and autistic 12- to 14-y-olds’ thresholds revealed no significant difference [*t*(21) = 0.28, *P* = 0.784].

### Neurotypical Children Systematically Integrate Social Influence into Perceptual Decisions Around 12 y of Age.

To investigate the effect of social influence on perceptual decisions, we then asked children to make perceptual judgments about the rotation direction of the SFM cylinder after being exposed to the response (advice) of either an age- and gender-matched peer or a gender-matched adult (visual task with social influence; Fig. 2*B*). First, we examined the behavioral response data statistically for changes with sensory evidence, gender, social influence direction, and age (*SI Appendix*, Table S1). Gender had no effect on responses for any age or experimental group (ANOVA; *P* = 0.079). But sensory evidence (binocular disparity) strongly affected judgments about the direction of cylinder rotation [ANOVA; *F*(6,119) = 152.40, *P* < 0.001], underlining children’s sensitivity to the visual task. Social influence in the form of peer advice had a significant effect on 6- to 8-y-olds [ANOVA; *F*(1,23) = 9.61, *P* < 0.002], but adult social influence did not. A post hoc test revealed that for ambiguous cylinder stimuli (bistable direction of rotation), the youngest children surprisingly tended to respond in the direction opposite to social influence rather than conform (Wilcoxon rank sum; *P* < 0.0001; binocular cylinder disparity of 0°; Fig. 3 *A* and *B*, *Top*). There was no significant effect of social influence at any other binocular disparity for this age group. For 9- to 11-y-olds, we found no significant effect of social influence on responses, whether the advisor was an adult or a peer [ANOVA; peer *F*(1,13) = 0.81, *P* = 0.370; adult *F*(1,16) = 3.36, *P* = 0.067] (Fig. 3 *A* and *B*, *Middle*). However, the responses of 12- to 14-y-olds were strongly affected by social influence [ANOVA; peer *F*(1,15) = 129.53, *P* < 0.001; adult *F*(1,24) = 641.14, *P* < 0.001]. When assessed stimulus by stimulus, this effect was evident at almost every binocular cylinder disparity and always in the direction of social influence (Wilcoxon rank sum; *P* < 0.0001) (Fig. 3 *A* and *B*, *Bottom*).

To assess whether integration of social influence resulted in a consistent bias in perceptual reports, cumulative Gaussian functions were fitted to the normalized, pooled behavioral responses for each age and experimental group (Fig. 3). One function was fitted to behavioral responses when the social influence of the peer or adult was “left,” and another when it was “right.” Responses of the younger two neurotypical age groups were equally well fit with a single cumulative Gaussian function (i.e., not separating responses between social influence left and right) as with two separate functions, which suggests that their judgments were not biased in the direction of social influence <[6 to 8 y, adult advice: adjusted R-squared (RSQ) = 0.956 and 0.979, *P* = 0.550; peer advice: adjusted RSQ = 0.932 and 0.941, *P* = 0.480] and (9 to 11 y; adult advice: adjusted RSQ = 0.975 vs. 0.977, *P* = 0.720; peer advice: adjusted RSQ = 0.936 vs. 0.948, *P* = 0.610)>. For 12- to 14-y-olds, responses were better described by fitting two functions, which were allowed to differ by social influence direction, as compared to one (adult advice: adjusted RSQ = 0.983 for two curves vs. adjusted RSQ = 0.535 for one curve, *P* < 0.0001; peer advice: adjusted RSQ = 0.972 for two curves vs. adjusted RSQ = 0.788 for one curve, *P* < 0.0001).

**Fig. 4.**
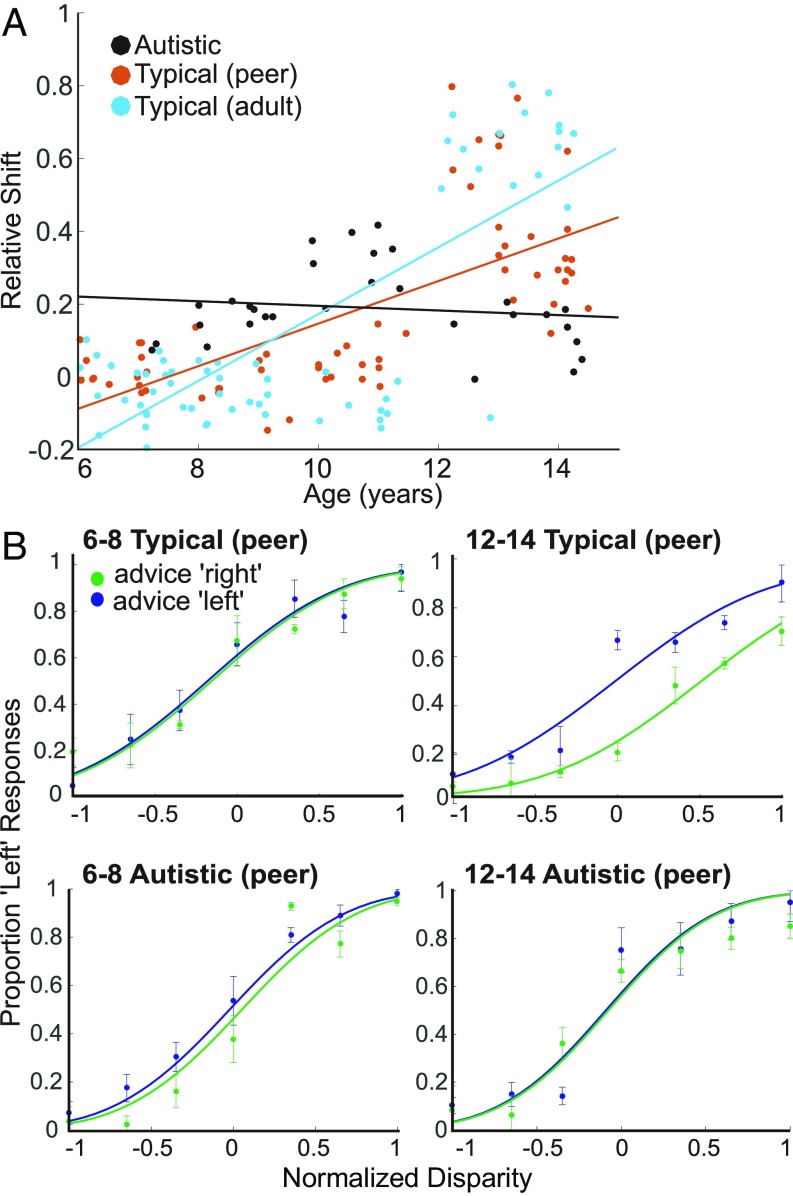
Individual participants’ psychometric functions under leftward and rightward social influence. (*A*) Each data point plots the relative shift (bias) between the two fitted Gaussian functions for one participant under the two different directions of social influence against the participants age at the time of testing. The shift was measured after normalizing each participant’s tested disparity range to ±1. Most neurotypical children below the age of 11 y showed no or only a small bias in their response function in the direction of social influence, in contrast to neurotypical children aged 12 to 14 y. For both neurotypical experimental groups, there was a moderate positive linear relationship between age and shift size (peer correlation coefficient *r* = 0.69; adult *r* = 0.76). In autistic children, an earlier, weak bias to social influence appeared reduced in the older children, which was reflected in the weakly negative linear relationship between age and shift size (*r* = −0.14). (*B*) Representative individual psychometric functions from the youngest and oldest age groups for matched neurotypical and autistic participants. Error bars show 95% confidence intervals. All individual functions are shown in *SI Appendix*, Fig. S1.

We also fitted paired functions to individuals’ psychometric data (see *SI Appendix*, Fig. S1 for individual fits and *SI Appendix*, Table S2 for goodness of fit). For comparisons across individuals with different stereo thresholds, normalized disparity values were used, with the largest disparities set to ±1 for each child. Calculating the shift between rightward and leftward curves (with the slope constrained to be the same) quantifies the bias, and a significant positive shift indicates a bias in the direction of social influence (Fig. 4). For neurotypical children, the perceptual judgments of 6- to 8-y-olds did not show a significant bias by social influence whether provided by an adult or by a peer [mean shift of 0.008 normalized disparity, *t* test; *t*(48) = 1.47, *P* = 0.150], which was similar for 9- to 11-y-olds [mean shift = 0.063 normalized disparity, *t* test; *t*(32) = 1.05, *P* = 0.300]. However, the responses of 12- to 14-y-old neurotypical children showed a large, significant mean shift of 0.419 (normalized disparity) in the direction of social influence [*t* test; *t*(42) = 14.96, *P* < 0.001]. For 12- to 14-y-olds, social influence consistently shifted perceptual judgments in the social influence direction, regardless of the type of advisor.

Overall, the younger neurotypical children showed no consistent directional bias in favor of social influence. In contrast, perceptual decisions of 12- to 14-y-olds showed a strong, consistent biasing effect of social influence on visual stimuli with varying strengths of sensory evidence, indicating systematic integration of social influence.

### Sensitivity to Social Influence but Less Systematic Integration in Perceptual Decisions by Autistic Children.

We tested the same perceptual decision-making paradigm using peer advice (Fig. 2*B*) in a group (*n* = 30) of autistic children (ages 6 to 8, 9 to 11, and 12 to 14 y). When we compared their results to those from age- and IQ-matched neurotypical children in the previous section, we found a significant difference in behavioral responses between autistic children and the matched neurotypical children (diagnosis) [ANOVA; *F*(1,88) = 7.91, *P* = 0.005] and a significant interaction of diagnosis and social influence [*F*(2,88) = 11.447, *P* < 0.001]. As for neurotypical children, gender had no significant effect on the responses of autistic children (ANOVA; *P* > 0.388). But we found a significant effect of social influence (advice) on responses for autistic children in all age groups (ANOVA; *P* < 0.001) (*SI Appendix*, Table S3). When the responses of autistic children were tested post hoc at different levels of visual evidence, 6- to 8-y-olds’ responses were biased in the direction of social influence for ambiguous, zero-disparity stimuli, but only at two of six nonzero binocular disparities (Wilcoxon rank sum; *P* < 0.0001) (Fig. 3*C*, *Top*). The responses of 9- to 11-y-olds went likewise with social influence at just two disparity levels (Wilcoxon rank sum; *P* < 0.0001) (Fig. 3*C*, *Middle*). Unlike neurotypical children of the same age, younger autistic children appeared to conform weakly to social influence. For 12- to 14-y-olds, we found little evidence of consistent social bias, with significant responses in favor of social influence for ambiguous stimuli but in the opposite direction at one nonzero disparity (Wilcoxon rank sum; *P* < 0.0001) (Fig. 3*C*, *Bottom*).

We then examined the effect of social influence on individual psychometric functions by fitting pairs of cumulative Gaussian functions that could differ in their horizontal offset with social influence direction. Psychometric functions described the data consistently well, with individual adjusted RSQ values ranging from 0.82 to 0.99 (see *SI Appendix*, Table S2 for summary). While mean RSQ was slightly higher for neurotypical (0.94) than for autistic children (0.92), their distributions did not differ (*F* test; *P* = 0.075). Unlike age- and IQ-matched neurotypical children, perceptual judgments of 6- to 8- and 9- to 11-y-old autistic children showed a small, significant shift in the direction of social influence [*t* test; 6 to 8 y: shift = 0.14 normalized disparity, *t*(8) = 8.34, *P* < 0.001; 9 to 11 y: shift = 0.29 normalized disparity, *t*(10) = 10.46, *P* < 0.001] (Fig. 4). Autistic adolescents (12- to 14-y-olds) showed a very small but significant shift of 0.03 (normalized disparity) [*t* test; *t*(9) = 2.90, *P* = 0.047] (Fig. 4). A direct comparison of individual psychometric shifts with peer social influence for autistic and matched neurotypical participants showed that only age group had a significant main effect (ANOVA; age group *P* < 0.001; gender *P* = 0.860; diagnosis *P* = 0.421). This effect was qualified by a significant interaction between age and diagnosis [ANOVA; *F*(2,79) = 76.52, *P* < 0.001], but no other significant interactions. Comparing shifts for the matched autistic and neurotypical older children alone (12- to 14-y-olds) showed a significant social influence bias in the autistic compared with the neurotypical children [ANOVA; *F*(1,25) = 50.32, *P* < 0.001]. There were also significant differences in shift for the two younger age groups, but in the reverse direction, with younger autistic children more affected by social influence than their neurotypical peers [6- to 8-y-olds: *F*(1,36) = 46.70, *P* < 0.001; 9- to 11-y-olds: *F*(1,24) = 142.30, *P* < 0.001].

**Fig. 5.**
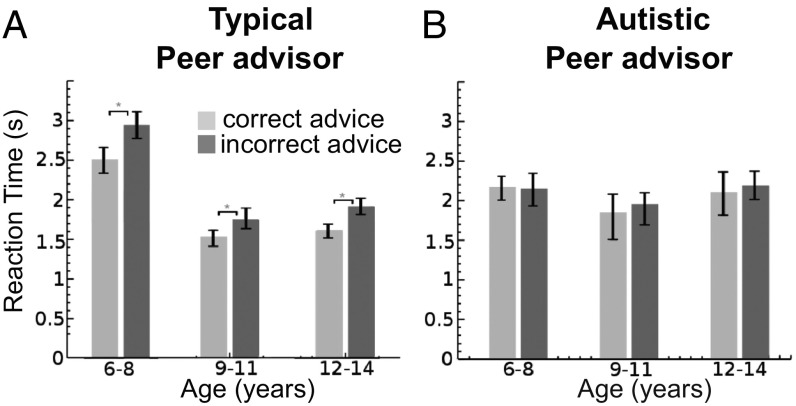
RTs for conforming with peer social influence for matched neurotypical and autistic children. (*A*) Median RTs for conforming decisions of neurotypical children were significantly faster when social influence direction was correct rather than incorrect. *P < 0.05, Wilcoxon rank sum, Bonferroni corrected; error bars show 95% confidence intervals. Advisor type did not have a significant effect on RT [ANOVA; *F*(1,165) = 2.91, *P* = 0.086]. (*B*) For autistic children, there was no significant difference in median RTs for conforming to correct or incorrect peer advice. Overall, an autism diagnosis did have a significant effect on RTs [ANOVA; *F*(1,165) = 4.97, *P* = 0.027]. For detailed RT distributions, see *SI Appendix*, Fig. S2.

The potentially different effects of social influence on perceptual decisions for autistic and neurotypical children were further underscored when we examined RTs. Across all neurotypical age groups, incorrect advice slowed down judgments relative to those made when the advice was correct with respect to the physical properties of the visual stimulus. In contrast, autistic children did not show this effect at any age (Fig. 5) and, when further broken down by disparity and social influence direction (*SI Appendix*, Fig. S2), autistic children showed this effect only weakly at the youngest age.

Inspecting visual task performance, a comparison of thresholds obtained with and without social influence for matched autistic and neurotypical children revealed that all participants performed worse under social influence [ANOVA; *F*(1,134) = 30.18, *P* < 0.001], but diagnosis was not a significant factor [ANOVA; *F*(1,134) = 1.93, *P* = 0.168]. This suggests that participants could not selectively integrate “useful” social influence. Disparity thresholds obtained under social influence differed significantly by both age and diagnosis [ANOVA; age *F*(2,83) = 3.232, *P* = 0.045; diagnosis *F*(1,83) = 7.831, *P* = 0.006] with a significant interaction [ANOVA; *F*(2,83) = 25.917, *P* < 0.001]. Notably, the oldest autistic children had lower disparity thresholds under social influence than the oldest neurotypical children [*t* test; *t*(25) = −7.539, *P* < 0.001], suggesting overall that the oldest autistic children performed better on the visual task than their neurotypical counterparts because they were less affected by social influence.

While we find a clear developmental trajectory of increasing social influence bias in neurotypical children, with a strong, systematic bias emerging around early adolescence, our data indicate potential differences in social information integration between neurotypical and autistic children.

### Drift Diffusion Model Suggests Changes in Sensory Processing with Social Influence.

**Fig. 6.**
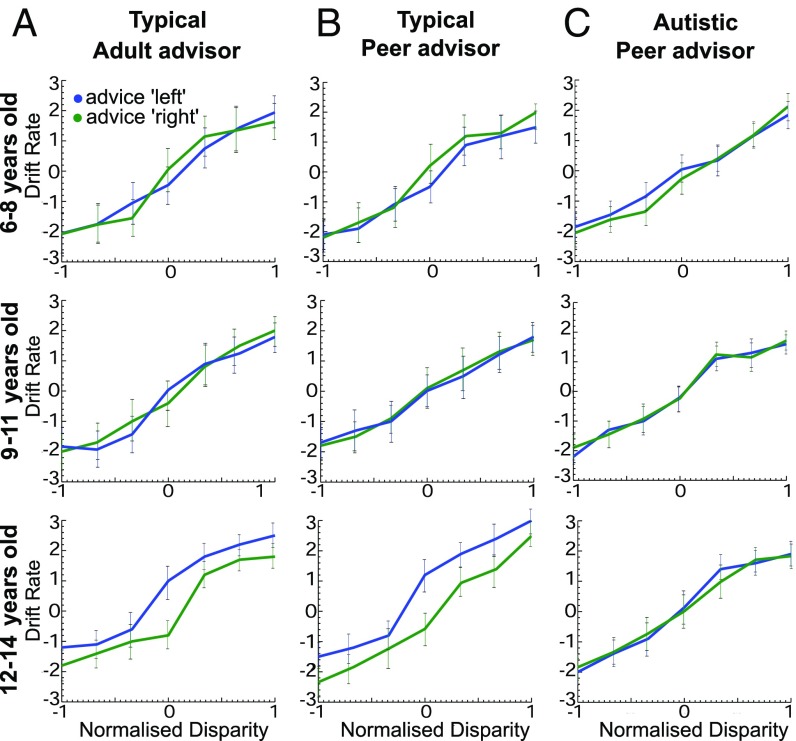
Drift rates under social influence for neurotypical and autistic children. (*A*) Drift rates were estimated for each stimulus disparity and social influence direction using a model in which drift rate and starting point were allowed to vary with social influence direction and with stimulus disparity. Median drift rates are plotted against normalized cylinder disparity for neurotypical children judging cylinder rotation under social influence from a gender-matched adult. As expected, median drift rates vary with the level of, and in the direction of, stimulus disparity at all ages, but only the 12- to 14-y-olds display a distinct deviation of drift rates in the direction of social influence. A positive drift rate indicates drift in the leftward response direction, and vice versa. (*B*) Median drift rates are shown for neurotypical children who made perceptual decisions under social influence from an age- and gender-matched peer. Again, only the 12- to 14-y-olds show a distinct deviation of drift rates in the direction of social influence. (*C*) Median drift rates for autistic children who were advised by an age- and gender-matched peer showed no systematic bias with social influence for any age group. Autistic children matched the neurotypical group in *B* in IQ. All conventions as in *A*. Error bars show 95% confidence limits.

To investigate the potential mechanisms that drove perceptual decisions in the children who took part in our study, we applied a computational drift diffusion model to our behavioral data collected under social influence. When allowing the model’s drift rate to vary in an unrestricted manner with cylinder disparity, drift rate had a clear relationship with sensory stimulus strength and direction, as predicted by previous literature (25, 42). This was evident across all age groups for neurotypical children and autistic children (Fig. 6). When we investigated the effect of social influence on different parameters of the models, we found that the best-fitting model in all cases required both parameters—drift rate, *v*, and starting point, *z*—to vary with advice direction (Model 4; Table 2). Model 4 across all age groups and conditions provided a good fit for both the RT distributions and the actual responses (*SI Appendix*, Figs. S3 and S4). For the best-fitting Model 4, neurotypical 12- to 14-y-olds showed a consistent bias in drift rate in favor of social influence across all levels of stimulus strength, unlike other neurotypical or autistic children (Fig. 6). The importance of the drift rate variable for this group was supported by the observation that for them, but not for any other age group and condition, changes in drift rate with social influence (Model 3) appeared to describe our data better than changes in starting point alone (Model 2) (*SI Appendix*, Figs. S3 and S4).

**Table 2.** BIC values for drift diffusion models

Model	Neurotypical children			Autistic children		
	6–8 y old	9–11 y old	12–14 y old	6–8 y old	9–11 y old	12–14 y old
1 (no effects)	12238.33	13481.52	12485.31	12093.95	11452.89	11946.77
2 (*z* only)	3534.5	4258.14	7841.21	4411.13	3948.38	4194.82
3 (*v* only)	6131.11	6927.81	4291.11	8474.41	7819.93	8733.9
4 (*v* and *z*)	**2984.53**	**3148.14**	**2918.18**	**2983.84**	**2751.88**	**3145.11**
5 (unrestricted)	11172.03	10029.23	10991.1	10938.52	10088.03	10947.82

For Model 1, no parameter was allowed to vary with social influence direction. For Models 2 and 3, either starting point, *z*, or drift rate, *v*, was allowed to vary with social influence. For Model 4, *v* and *z* were both allowed to vary with social influence, while other parameters were not. Model 5 was unrestricted. A lower BIC value indicates a better fit. For all groups, Model 4 showed the best fit (values in bold type). Models 2 and 3 appeared to perform differently for 12- to 14-y-old neurotypical children in contrast to all other groups.

**Fig. 7.**
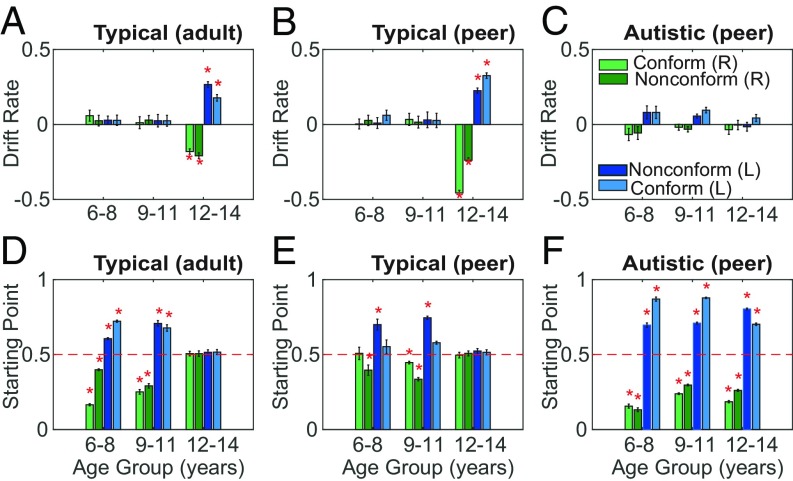
Drift rates and starting point estimates by social influence direction and conformity for the best-fitting drift diffusion model. (*A*) For neurotypical children aged 12 to 14 y, median drift rates systematically changed with the direction of adult social influence, whether they conformed or not. No such change in drift rate is apparent for younger children. Error bars show 95% confidence limits; an asterisk denotes a significant deviation from zero (Wilcoxon rank sum, *P* < 0.00001, Bonferroni corrected for significance at *P* < 0.05). (*B*) A similar pattern of drift rate changes with social influence direction is apparent for 12- to 14-y-old neurotypical children advised by a peer. The effect on drift rate appeared stronger when children conformed with advice. Conventions as in *A*. (*C*) For autistic children, we found no significant changes of drift rate with social influence direction or with conformity. Conventions as in *A*. (*D*) When estimating starting points by social influence direction and level of conformity with an adult advisor, we found that the two younger age groups of neurotypical children showed a significant deviation from the midpoint in the direction of social influence, but 12- to 14-y-olds showed no significant bias in starting point. Error bars denote 95% confidence limits; an asterisk shows a significant deviation of the starting point from 0.5 (Wilcoxon rank sum, *P* < 0.00001, Bonferroni corrected for significance at *P* < 0.05). (*E*) Neurotypical children showed a very similar pattern of starting point bias with age when advised by a peer or by an adult (*D*). However, the early bias in starting point appeared less strong. Conventions as in *D*. (*F*) Across all age groups, autistic children showed a strong bias in the starting point. Conventions as in *D*. For parameter estimates for Model 2 (starting bound only) and Model 3 (drift rate only), see *SI Appendix*, Fig. S5.

We also assessed the parameters drift rate and starting point by level of conformity and with social influence direction for Model 4. For 12- to 14-y-old neurotypical children, the drift rate parameter increased significantly in the direction of social influence and with conformity (regardless of whether the advisor was a peer or an adult) (Fig. 7 *A* and *B*) [ANOVA; *F*(1,41) = 169.00, *P* < 0.001]. In contrast, autistic children showed no consistent changes in drift rate parameter with advice or conformity (Fig. 7*C*) [ANOVA; *F*(2,29) = 0.73, *P* = 0.483], despite showing some behavioral conformity with social influence. Their drift rates were more comparable to those of neurotypical children between 6 and 11 y old (Fig. 7 *A* and *B*), with no effect of social influence [ANOVA; *F*(1,65) = 0.25, *P* = 0.618]. Generally, age had a significant effect on drift rate changes with social influence for neurotypical children [*N*-way ANOVA; *F*(2,124) = 7.95, *P* < 0.001]. Between the oldest autistic group and the matched neurotypical group, there was also a significant difference in drift rate [ANOVA; *F*(1,26) = 6.31, *P* < 0.001], which was not detected with all age groups included [*F*(1,89) = 0.67, *P* = 0.251]. Post hoc comparisons show that only the oldest neurotypical group is significantly different in drift rates to any other group (Tukey; *P* < 0.001). This supports the possibility that drift rates between the younger neurotypical groups were not dissimilar to those found in all autistic groups.

In contrast to drift rates, starting point values obtained for the best-fitting model significantly deviated from the midpoint (0.5) between the two decision bounds for autistic children in all three age groups (Fig. 7*F*). The direction of the starting point deviation was always in the direction of social influence. For the 6- to 8-y-old and the 9- to 11-y-old neurotypical children, starting point also showed deviation from the midpoint (Fig. 7 *D* and *E*). However, for the oldest neurotypical group, who showed the strongest social conformity in perceptual decisions, starting point did not significantly deviate from the midpoint. When directly comparing matched autistic and neurotypical children, diagnosis and age both affected the starting point [ANOVA; diagnosis *F*(1,89) = 472.90, *P* < 0.001; age group *F*(2,89) = 4.84, *P* = 0.010], with a significant interaction [ANOVA; *F*(2,89) = 51.55, *P* < 0.001]. Thus, large changes in the starting point in the direction of social influence may reflect the response bias of the younger autistic children to social influence relative to their neurotypical peers. This decision bias might be achieved by different processes than in the 12- to 14-y-old neurotypical children, whose data show, in contrast, a large change in drift rate parameter.

## Discussion

Using a single advisor and a perceptual stereomotion task, we examined the developmental trajectory of social influence integration in neurotypical children between the ages of 6 and 14 y and in a small cohort of age- and IQ-matched autistic children. Behavioral results and those from drift diffusion modeling show a clear developmental change for neurotypical children in behavior and information processing around the age of 12 y. From this age, whether advised by a peer or an adult, neurotypical children showed perceptual decisions strongly and systematically biased by social influence across all levels of sensory stimulus strength. In contrast, the social influence response bias evident in the decisions of younger autistic children was weak and appeared to diminish with age. Drift diffusion models exhibited comparable results for autistic children and neurotypical 6- to 11-y-old children, but not for the neurotypical adolescents. Their significant changes in drift rate with social influence could indicate effects on sensory processing, in contrast to a decision bias indicated for the autistic children. Our results clearly show that the developmental trajectory of the mechanism for social influence integration in neurotypical children becomes most prominent in early adolescence. While the autistic cohort was small, our results suggest that there might be a divergence in the neurodevelopment of the social brain at this stage.

### The Development of Social Influence in Neurotypical Children.

We observed consistent social influence in the oldest age group of neurotypical children that we tested, regardless of advisor type. While a number of previous studies have provided evidence for sensitivity to social influence during childhood (e.g., refs. 14, 43, and 44), our study found a relatively later onset of social influence on perceptual decisions. When comparing social influence studies, it is important to consider that different scenarios are often used to induce social influence and that there are differences in the nature and difficulty of the tasks children are asked to complete. The studies mentioned above were mainly based on social influence through majority opinions, which probably induce both normative and informational social influence (45). The effect of normative influence, or group pressure, is susceptible to variation in the environmental setting, the nature of giving the response, and group composition (1, 2, 46). All of these factors can affect the experience of social influence and could explain the discrepancy between our results and those of previous studies. In contrast, our paradigm was designed to minimize group pressure and provide informational social influence through the use of a single gender-matched advisor, controlled video sequences, and a stereo-blind experimenter who could not discriminate the rotation direction of the cylinder stimulus. Given these experimental manipulations, we would expect biasing effects to be weaker, but this should allow us to probe the mechanisms of informational social influence more selectively.

Another important question is whether the effects we observed were specific to the “social” aspect of the advice. There is evidence for development of adult-like visual cue integration around the age of 10 y (47). All of the children in our study performed at adult-like levels in the visual task alone, which required the combination of motion and binocular depth cues to resolve the cylinder percept. But this is different from cue integration, which adds information from two cues about the same feature value, improving task sensitivity (47). The integration of social influence might be a different process again, because behaviorally, the main effect is a bias in responses. We did not include blocks with an abstract advice cue, in part because removing all social meaning, even from abstract cues, is fraught with problems, as people often attribute human features to machines and even abstract objects (48–50). At an anecdotal level, children in our study would at times interact with the advisor by addressing the on-screen advisor, offering comments about the advisor and how reliable they were, thus treating the on-screen advisor as another person. Furthermore, in common with many earlier social influence studies, we found that the effect of social information on perceptual decisions was most apparent when sensory information was ambiguous (14, 51, 52), despite the fact that observers cannot readily distinguish ambiguous and unambiguous SFM figures as used here (53).

When children were making perceptual decisions about stereomotion figures, social influence biased perceptual decisions systematically across different stimulus levels (ambiguous and unambiguous) from early adolescence. This seems at odds with earlier research indicating that conformity to unambiguous stimuli decreased between the ages of 3 and 10 y (14, 54), while across the same period, conformity to ambiguous stimuli increased (14, 52). Considering these studies together with our data suggests that, although normative influence/group pressure appears to exert an effect at an earlier age, informational social influence may work through a different mechanism, which becomes more prominent later in development. This possibility is supported by the differential effects of social influence on the two key variables of the drift diffusion model at different ages with our paradigm: The neurotypical 12- to 14-y-olds’ strong variations in drift rate for social influence suggest that the effects of social influence may be due to biasing sensory processing (perhaps weighting sensory stimulus representations by social information). In contrast, children under the age of 12 y tended to show stronger deviations in the starting point for different advice directions, suggesting an “intentional” decision bias without an effect on sensory processing (as discussed in refs. 25 and 55). Over the course of development, children may become genuinely perceptually biased by social information, which needs to be confirmed with other perceptual decision paradigms in the future.

### The Development of Social Influence in Autistic Children.

A caveat to our results from autistic children is the small number of participants. Because autism is a heterogeneous condition, it can be difficult to generalize behavior, even from larger samples. While our results cannot be generalized to autistic populations with additional intellectual disabilities and/or limited spoken communication, for the participating group of autistic children, results reached statistical significance and individual-level biases were found to be consistent. Indeed, autistic children showed similar perceptual decision behavior across all age groups, with a weak response bias in favor of social advice. The absence of a strong, systematic social bias in the 12- to 14-y-old autistic children fits well with previous research highlighting reduced weighting of social information. For instance, many autistic children and adults do not attend to socially salient aspects of visual information (56, 57) and are less responsive to notions of reputation (58, 59) or flattery (60) compared with their neurotypical counterparts. Our behavioral and computational results suggest a distinct developmental time point and mechanism that appear to differ. Drift diffusion results suggest that changes in sensory processing that might systematically bias neurotypical adolescents’ perceptual choices do not emerge in autistic children at the same age. In contrast, the younger neurotypical and autistic children had starting point biases, suggesting that when their decisions were socially influenced, it might rather have been the product of intentional decision bias. It is important to note, however, that being less susceptible to social influence resulted in the older autistic group performing more accurately than their neurotypical counterparts. These findings are consistent with alterations in a proposed cognitive mechanism for filtering incoming sensory evidence based on prior expectations, which is thought to be attenuated in autistic people (23, 24).

Psychometric functions for some autistic children appeared noisier under social influence. While there is some evidence of increased internal noise in the processing of perceptual stimuli for autistic children (61, 62), this is contentious (63, 64). Our threshold data indicated that all children could perform the visual task without social influence to equivalent levels. The effect of social influence may have been distracting or confusing for autistic children. A recent paper suggested that higher levels of evidence-related noise led to decisions that were faster, less accurate, but more confident (28). This might potentially explain the appearance of a small bias in the autistic groups. It would be interesting to investigate confidence ratings of perceptual decisions for autistic and neurotypical children with this paradigm. Together, our data show that unlike neurotypical children, autistic children in our study do not show evidence of specific age-related changes in the mechanism of social influence integration into decision making.

### Potential Neural Substrates for Social Influence.

Our results not only indicate a specific developmental stage at which neurotypical and autistic children seem to diverge in how they process social influence for decision making, they also provide us with an opportunity to search for the neural substrate responsible for the underlying processes. As children develop, the connectivity between different brain regions changes. In neurotypical children, cortical gray matter and white matter increase between the ages of 4 and 20 y, peaking at 12 y for the frontal and parietal lobes (65, 66). Changes have been attributed to hormonal factors, especially with regard to puberty influencing both brain structure and function (67, 68). Our own results, and results from EEG and drift diffusion modeling in adults, converge on a model in which social influence biases already early sensory processing (6, 7, 69–71). Sensory signals that contribute to the reported percept for the stereomotion visual task in this study have been linked to primate visual area V5/MT, which is located on the posterior bank of the superior temporal sulcus (STS) in rhesus macaques (34, 35). Sensory processing in V5/MT is affected by contextual factors that can bias perceptual decisions like expected reward (72). In humans, the functionally homologous area, hMT+, lies usually in the inferior temporal sulcus, near the STS (73). In primates, cortical areas in and near the STS are thought of as a social network node, processing aspects of social behavior such as biological motion or facial expressions (74–76). MRI has shown in neurotypical children that gray matter in the STS increases throughout childhood and adolescence to the age of 16 y (66). Social sensitivity has been proposed to decline again from around 16 y (8)—the point at which gray matter in the STS region also seems to decrease.

The STS and the dorsal visual stream associated with it were previously implicated in perceptual atypicalities and specific difficulties in social cognition associated with autism (77–79). In autistic children, this region of interest shows opposite structural changes during development from those described above for neurotypical children [e.g., white matter structure was atypical in autistic children, particularly in the temporoparietal junction and the STS (80)]. Gray matter volumes were decreased in the posterior STS (81). These changes in both gray and white matter in social-specific regions, and their connections, might indicate reduced connectivity with visual regions nearby and sensorimotor structures. This could potentially contribute to the decreased social bias we have observed in older autistic children. Studies of brain structure and connections of these regions at higher spatial resolution should be linked to behavioral assessment of social influence in the same individuals. Given the direct functional homologies of key cortical areas for perceptual and social behavior across primates and the ability of macaques to carry out perceptual tasks and to show social learning (5, 82–84), it should be feasible to study underlying mechanisms of social influence integration directly at the level of single neurons.

In conclusion, we identify a distinct developmental trajectory for the neurocognitive integration of social influence. By the age of 12 y, a mechanism appears to emerge in neurotypical children that allows the opinion of others to systematically bias sensory processing. Autistic children may not show this change, which might be correlated with differences in brain architecture around the STS region.

## Materials and Methods

Procedures were conducted in accordance with the Declaration of Helsinki (2008), and ethical approval for the study was granted by the Research Ethics Committee at the UCL Institute of Education, University College London (FCL 260). Parents or guardians of all children provided written informed consent before their child’s participation in the study. Children also gave their assent to take part.

### Participants.

We included behavioral data from 125 typical children and 30 children on the autism spectrum between the ages of 6 and 14 y in our analysis. All children in the autistic group had received an independent clinical diagnosis of an autism spectrum condition according to *International Statistical Classification of Diseases and Related Health Problems* (85) or *Diagnostic and Statistical Manual of Mental Disorders* (86) criteria and met the threshold for autism spectrum (a score of or exceeding 15) on the Social Communication Questionnaire (SCQ) (87) and the Autism Diagnostic Observation Schedule, Second Edition (88, 89) (*SI Appendix*, Table S4). All autistic children had an IQ in at least the average range and were thus considered to be cognitively able. These children were recruited through community contacts.

All children were divided into three age groups and according to condition and experiment. The autistic children were divided into age groups as follows: 6 to 8 y: *n* = 9 (one female), 9 to 11 y: *n* = 11 (two females), and 12 to 14 y: *n* = 10 (three females). One comparison group of 61 neurotypical children was matched to the group of autistic children in terms of chronological age and intellectual functioning (*SI Appendix*, Table S4). They were divided into age groups of 6 to 8 y: *n* = 29 (12 females), 9 to 11 y: *n* = 15 (9 females), and 12 to 14 y: *n* = 17 (10 females). They took part in the same experiment as the autistic children. Data were also collected for a separate set of 64 neurotypical children on the same visual task but in a different social condition—with an adult as advisor, not a peer. These children were also divided into three age groups: 6 to 8 y: *n* = 20 (11 females), 9 to 11 y: *n* = 18 (12 females), and 12 to 14 y: *n* = 26 (20 females).

All participants included in this study passed a test for normal or corrected-to-normal visual acuity using a Snellen chart (minimum acuity 6/9 with correction) and showed evidence of stereovision with a static random dot stereogram TNO test [TNO test for stereoscopic vision (Lameris Ootech) using plates V-VII with a minimum threshold of 240 s of arc]. Before the social experiment, the majority of participants engaged in a short threshold test using the same stereomotion visual stimulus but without social influence (Fig. 2*A* and Table 1). Establishing a binocular disparity threshold allowed the application of perithreshold disparities to obtain a smooth psychometric function from individual participants. Because of time constraints, we could not obtain separate stereo thresholds for 22 participants in the neurotypical control group (6 to 8 y: *n* = 11; 9 to 11 y: *n* = 6; 12 to 14 y: *n* = 5). To ensure that these children also carried out the visual task adequately, we excluded in the analysis participants who failed to obtain 85% correct responses for the largest binocular disparity used or who showed a bias of more than 80% toward either response for ambiguous, zero-disparity visual stimuli. Based on these criteria, we excluded an additional 13 neurotypical children and 10 autistic children (three of whom were stereo-blind when assessed with the TNO test).

All the autistic children, their matched neurotypical comparison group, and 40 of the 64 adult-advised neurotypical group completed the Wechsler Abbreviated Scale of Intelligence – Second Edition (90) to index intellectual functioning and the SCQ as a measure of autistic symptomatology (ref. 87, *SI Appendix*, Table S4).

### Experimental Setup.

Data were collected at participants’ schools or homes, at the UCL Institute of Education, University College London, or at Oxford University. Visual stimuli were coded in MATLAB using the Psychtoolbox extension (91) on an Apple MacBook Pro (2.7 GHz Intel Core i7, Intel HD Graphics 4000 1024MB). They were displayed on an ELO surface capacitive touchscreen (model E653173, screen size 22 inches) with a resolution of 1280 × 1024 pixels at a frame rate of 60 Hz, in a darkened room. The viewing distance was 40 cm and participants wore red-blue glasses to enable them to see the anaglyph 3D signals.

### Visual Stimuli.

The visual stimulus was an SFM cylinder composed of two transparent surfaces with black and white dots on a midgray background. The dots moved in opposite directions with a sinusoidal velocity profile. The cylinder rotated through 90° of 360° over a period of 1 s (0.25 turns per second). Stimulus size was 6° × 6° of visual angle, with 125 black and 125 white dots randomly positioned on each cylinder, each dot subtending 0.2°. One percent of dots randomly died and were replotted in new, random positions on the cylinder for each frame. Cylinder surfaces could be separated in depth by applying different binocular disparities to the dots moving in opposite directions using anaglyph red-blue stimuli. The binocular disparity reported is measured from the center axis of the cylinder to the front; the sign gives the direction of rotation. There was a central white fixation point subtending 0.1° visual angle.

Before the experiment, participants were shown a Microsoft PowerPoint presentation introducing them to the cover story: They were instructed that they were taking part in a spaceship pilot training course and would judge the direction of spin of “black holes” to navigate a spaceship around them (*SI Appendix*, Fig. S6). Immediately before the social task began, they were introduced to their advisor via a separate PowerPoint presentation (*SI Appendix*, Fig. S7).

In the social influence conditions, a brief video clip (length 3,000 ms) of an advisor was shown before each visual stimulus presentation. For the autistic group and the matched group of neurotypical children, the advisor was both age and gender matched to the participant (the peer-advisor condition). For another neurotypical group, the advisor was gender matched to the participant but was an adult (the adult-advisor condition). The video was presented such that the advisor was facing the participant and looking into the camera. They delivered the same line in different 3-s videos: “The black hole is spinning [left/right].” There were 10 to 14 unique videos per advice direction (i.e., 20 to 28 videos of each advisor in total). All participants in an age group had the same gender-matched peer advisor or the same gender-matched adult advisor.

At the end of a block of trials, participants were shown a screen providing positive feedback to motivate them to continue with the task. There were 10 different, randomly chosen versions of feedback on a space-themed background, such as “Well done, you got the space ship home safely.”

### Experimental Tasks.

#### Measuring stereo acuity threshold.

Before engaging in the social influence condition, 132 of 155 children took part in a disparity threshold test with the same SFM stimulus as in the main experiment. This consisted of two blocks of 42 trials evenly distributed across seven binocular disparities for the SFM cylinder (typically ±0.03°, ±0.02°, ±0.01°, 0°), which were pseudorandomly interleaved. First, a central fixation point was presented for 500 ms, followed by an SFM cylinder for up to 2,000 ms (Fig. 2*A*). Participants had been instructed to respond at any point from stimulus onset as fast and as accurately as they could. Participants responded by pressing either to the left or right of the stimulus on the touchscreen (labeled “left” and “right” on the screen) to indicate in which direction the dots of the cylinder’s front surface were moving.

#### Left–right choice control.

To ensure that all children understood and could differentiate left and right before engaging in the experiment, they were asked to demonstrate with their finger which direction was “spinning left,” and which direction was “spinning right.” A subgroup of children [neurotypical 6- to 8-y-olds (*n* = 29), 9- to 11-y-olds (*n* = 30), and 12- to 14-y-olds (*n* = 17) and autistic 6- to 8-y-olds (*n* = 9), 9- to 11-y-olds (*n* = 11), and 12- to 14-y-olds (*n* = 10)] also took part in a brief computerized control experiment before the threshold task. They were asked to touch either the left or the right side of the screen (10 times per side, pseudorandomly interleaved) with the textual instruction “Please touch the [left/right] side of the screen.” All participants in this study could accurately and reliably differentiate between left and right (>95% correct).

#### Perceptual decision making with social influence.

The social influence task had the same basic trial structure as the visual threshold task, but an additional 3,000-ms social information “advice” video preceded the visual stimulus (Fig. 2*B*). Participants undertook 10 blocks of 21 trials across seven disparities and two types of social influence (left and right; 210 trials total). Depending on the stereo threshold estimated earlier, different ranges of SFM stimuli were used (high threshold: ±0.06°, ±0.04°, ±0.02°, 0°; mid threshold: ±0.03°, ±0.02°, ±0.01°, 0°; and low threshold: ±0.015°, ±0.01°, ±0.005°, 0°; the most common range used across all participants was the mid threshold). For unambiguous stimuli, advice was correct in two out of three trials. For ambiguous, 0° disparity SFM stimuli, the direction of advice was 50:50 left:right across trials. Different advice trials were pseudorandomly interleaved. Direction of response and RT data were recorded for each trial. An experimenter was in the room with the children as they did their task but was 3D stereo-blind and therefore did not have a 3D percept of the cylinder stimuli to be judged.

### Analysis.

#### Excluded trials.

Any responses taking longer than 8 s were excluded from further analysis. This was to include decisions in which a child might have struggled with a conflict between sensory information and social influence but to exclude trials in which the child failed to perform accurately due to distraction or attempting to engage with the experimenter [across all age groups, 24 trials (0.09%) were excluded, with a mean duration of 14.67 s].

#### Psychometric functions.

Psychometric functions were plotted for each individual participant, experimental condition, and social influence direction. Thresholds for binocular disparity were established by fitting the behavioral responses with a cumulative Gaussian function, using custom MATLAB scripts based on the MATLAB *cdfplot* function. When we pooled or compared results for different groups, we normalized each individual’s binocular disparity range to −1 to +1 (for smallest and largest disparity, respectively). To present group averages, we plotted the average behavioral response at each normalized stimulus disparity level. In the social condition, responses were separated by the direction of social influence (left or right) plotted over normalized disparity strength. A pair of cumulative Gaussian functions was fitted to each individual psychometric dataset (one function for each social influence direction) and the shift between the two fitted curves was measured when their slope was restricted to be the same. Goodness of fit of these functions, on average, exceeded adjusted RSQ of 0.91 for each experimental group (*SI Appendix*, Table S2).

The effect of social influence at individual disparities was tested in paired fashion using a nonparametric Wilcoxon rank sum test conducted in MATLAB (*ranksum* function) because data distributions were not normal (tested with the Kolgorov–Smirnov normality test). *P* values were corrected for multiple comparisons with the Bonferroni correction. An *N*-way ANOVA was applied in JASP (92) to test for the effect of social influence direction, age group, gender, and disparity on behavioral responses.

#### Drift diffusion model fitting.

Behavioral responses and RTs were fitted with a drift diffusion model in MATLAB using the Diffusion Model Analysis Toolbox (DMAT) (93), based on ref. 25. The model was fitted to group level rather than individual data, as large numbers of trials are required for accurate modeling (94), which would not have been possible to collect from individual children, given their age. By modeling the RT distributions under different conditions, the drift diffusion model predicts the parameters drift rate (*v*, the rate at which evidence accumulates) and starting point (*z*, the point from which the decision variable drifts) (Fig. 1). These two parameters can be used to make inferences about the potential mechanisms shaping the decision process (25, 26). To investigate the effect of social influence on model parameters, five models were tested. These models were chosen to investigate the two parameters that have been explicitly linked to central mechanisms involved in perceptual decision making:

Model 1: No effect of social influence allowed on any parameter.
Model 2: Starting point, *z*, only varies with social influence.
Model 3: Drift rate, *v*, only varies with social influence.
Model 4: Drift rate and starting point may vary with social influence.
Model 5: Unrestricted model—all parameters may vary with social influence.

In these models, all parameters were allowed to vary with stimulus disparity, including, additionally, boundary separation, *a*; nondecision time, T_er_; intertrial SD of drift rate, *eta*; range of starting point values, *sz*; and range of nondecision time, *st*. The values for these parameters are reported for the best-fitting model in *SI Appendix*, Table S5. Separately, we had used a similar set of models to investigate the effect of stimulus disparity (the strength of visual information given by the stimulus) on model parameters *v* and *z*, which confirmed the expected relationship between drift rate and stimulus disparity.

To compare the fit of different models, we used the Bayesian Information Criterion (BIC) (95). A lower BIC value corresponds to a better fit. The BIC tends to give worse fits with increasing model complexity (96, 97) and, as such, penalizes unrestricted models with a large number of varying parameters. A comparison of BIC scores between models with a paired *t* test (significant at a Bonferroni-corrected *P* < 0.05) identified the best fit for each case. Using the best-fitting model as determined by the BIC, the significance of nonzero drift rates and a starting point away from the midpoint were assessed under different advice and behavioral conditions (e.g., conforming with social influence left; not conforming with social influence left). Because data were not normally distributed, a nonparametric Wilcoxon rank sum (Bonferroni-corrected *P* < 0.05) was used.

To assess goodness of fit, we also compared qualitatively the RT distributions predicted by drift diffusion Models 2, 3, and 4 with actual RT distributions for different age groups. DMAT function “edfcdf” was used to overlay cumulative empirical and predicted RT distributions for each model (*SI Appendix*, Fig. S3). In addition, mean psychometric responses of participants in different age groups were fitted with predictions from best-fitting Model 4. The cumulative density functions were generated with the DMAT (*SI Appendix*, Fig. S4).

### Data Access.

Data and analysis scripts are available on request from the corresponding author. Because of the sensitive nature of children’s personal and clinical data, there might be restrictions on releasing individual raw data and we might have to consult the local ethical review process.

## Supplementary Material

Supplementary File
