# Supplementary material for: Developmental trajectory of social influence integration into perceptual decisions in children
Source: Proc Natl Acad Sci U S A. 2019 Jan 28;116(7):2713–22. doi: 10.1073/pnas.1808153116 (PMC6377450; doi:10.1073/pnas.1808153116)
Supplement: Supplementary File [file pnas.1808153116.sapp.pdf]

PNAS
www.pnas.org

Supplementary Information for

The development trajectory of social influence integration into perceptual decisions for children

Imogen Large[1*], Elizabeth Pellicano[2,3], Andreas Mojzisch[4], Kristine Krug[1]

Prof Kristine Krug
Email: kristine.krug@dpag.ox.ac.uk

This PDF file includes:

Figs. S1 to S7
Tables S1 to S5

www.pnas.org/cgi/doi/10.1073/pnas.1808153116

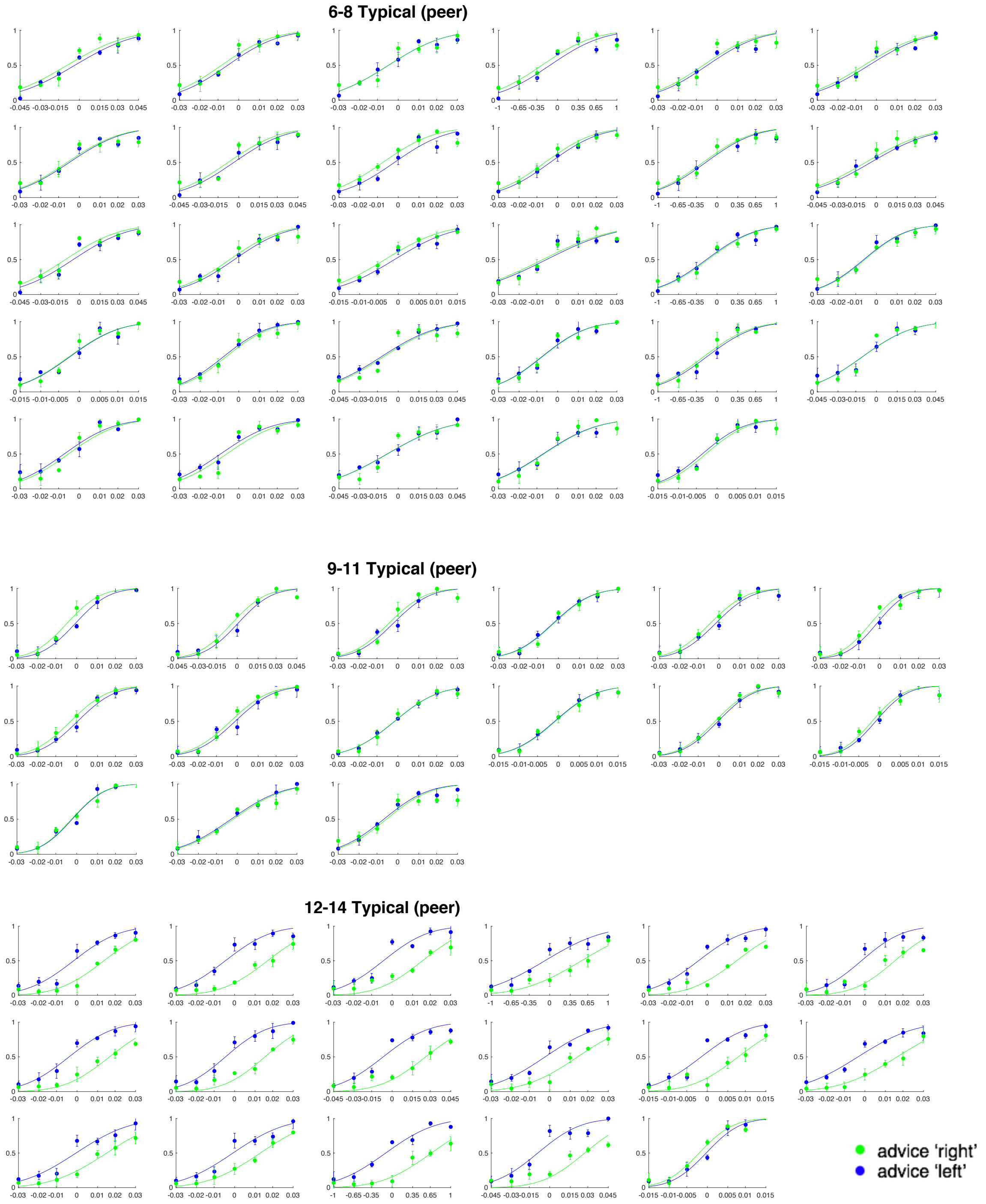

Fig. S1. A. Individual psychometric functions from the ‘peer advised’ neurotypical participants. Blue denotes perceptual choices for social influence to the ‘left’, green for social influence to the ‘right’. Error bars show 95% confidence intervals. The x-axis shows binocular disparity (positive values define leftward rotation), the y-axis proportion of ‘leftward’ choices.

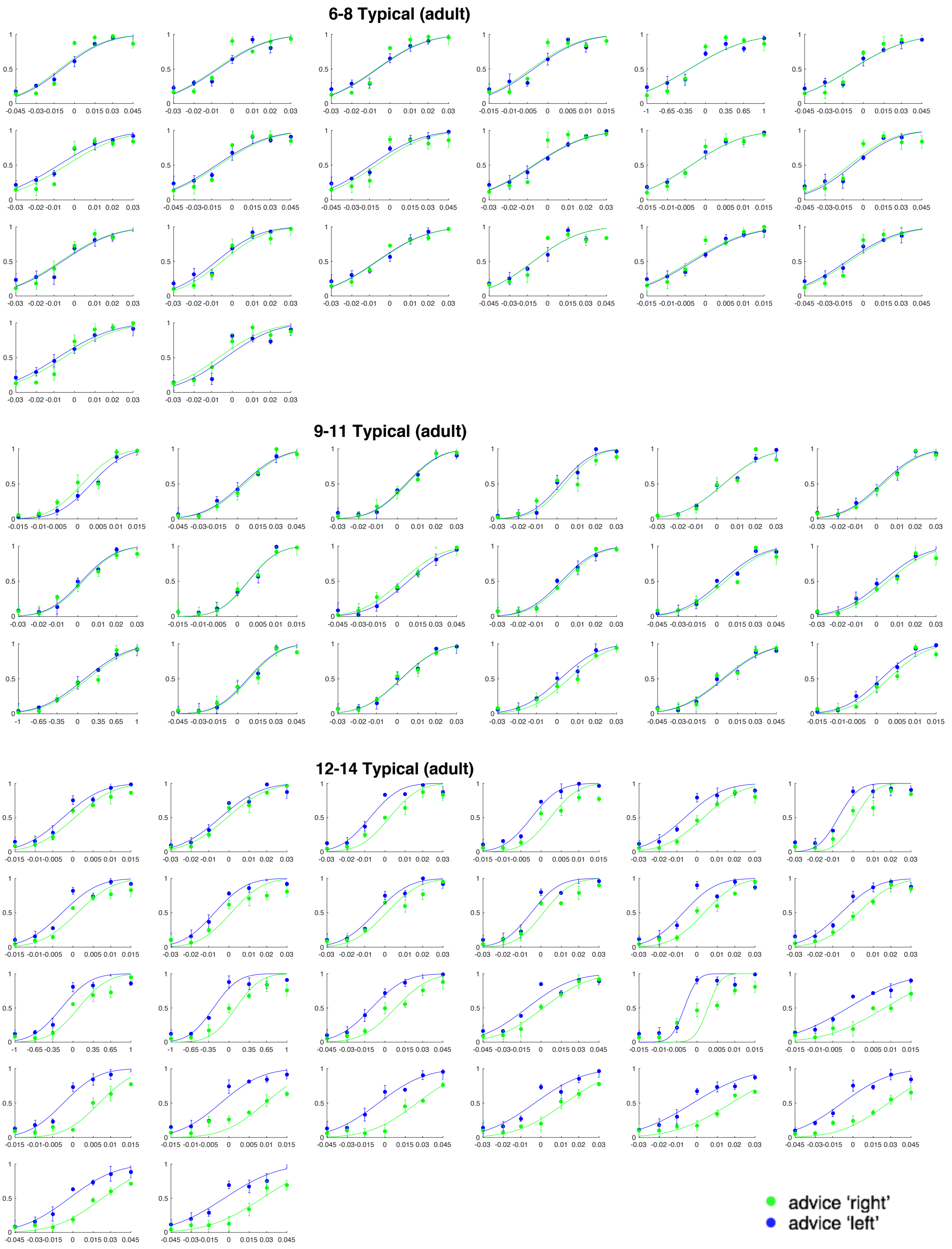

Fig. S1. B. Individual psychometric functions from the 'adult advised' neurotypical participants. Blue denotes perceptual choices for social influence to the 'left', green for social influence to the 'right'. Error bars show 95% confidence intervals. The x-axis shows binocular disparity (positive values define leftward rotation), the y-axis proportion of 'leftward' choices.

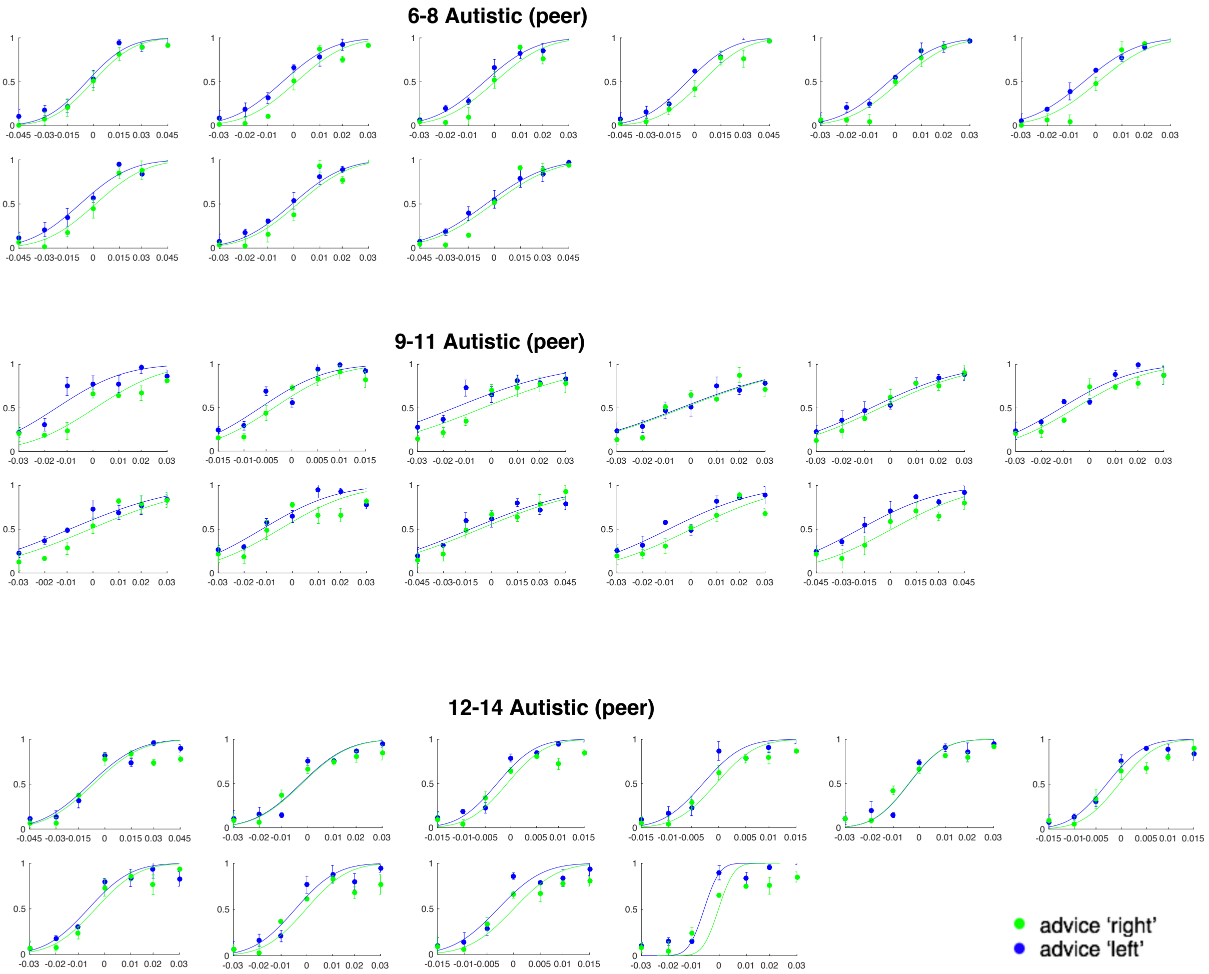

Fig. S1. C. Individual psychometric functions from the 'peer advised' autistic participants. Blue denotes perceptual choices for social influence to the 'left', green for social influence to the 'right'. Error bars show 95% confidence intervals. The x-axis shows binocular disparity (positive values define leftward rotation), the y-axis proportion of 'leftward' choices.

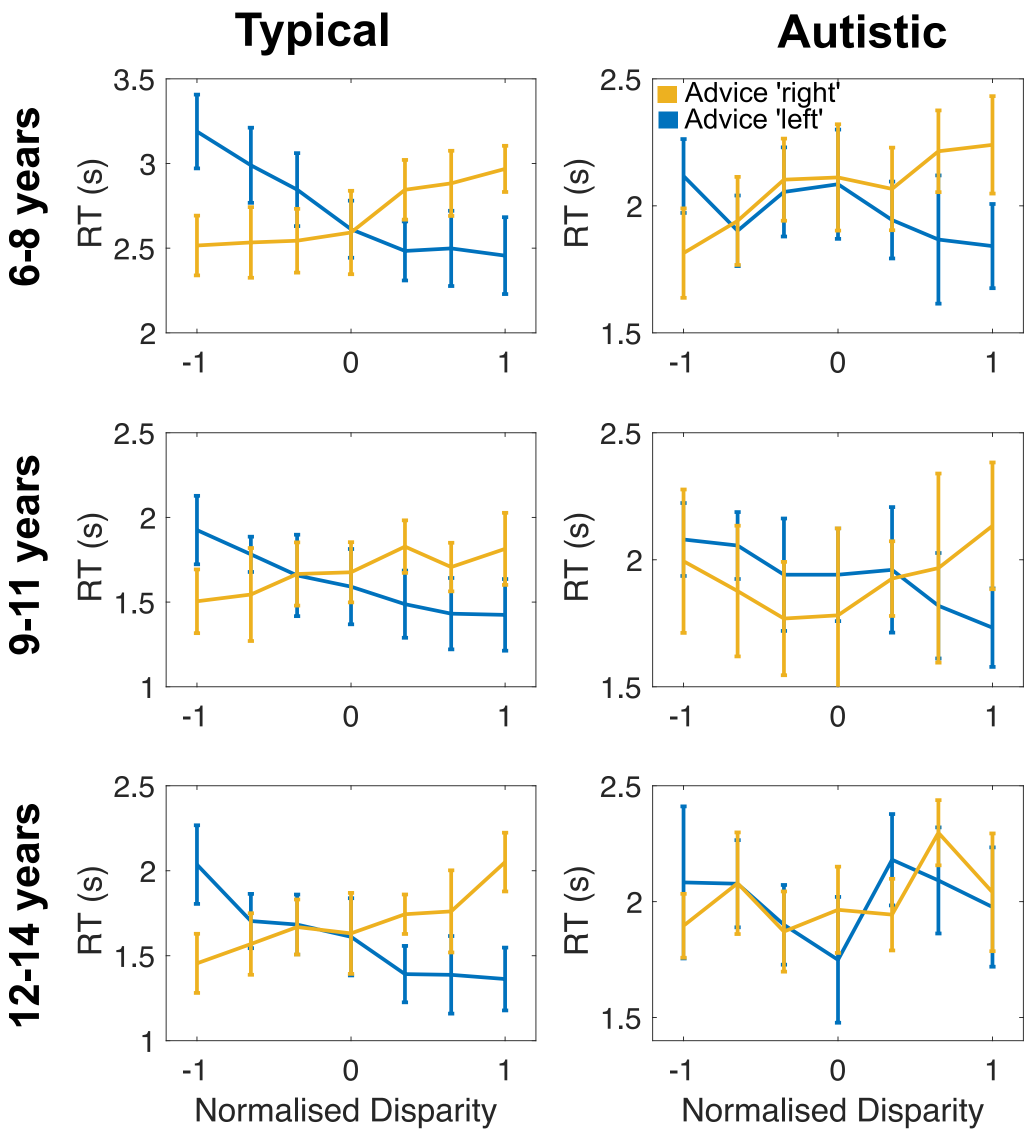

Fig. S2. Reaction time (RT) distributions for perceptual decisions when social influence (advice) was to the 'left' or to the 'right'. For neurotypical children, as expected, when social influence conflicted with sensory information in the form of binocular disparity, reaction times were slower. For autistic children, this pattern is weakly present in the youngest age group, but is not clearly discernible for older children. The RT data supports the evidence presented in Figure 5 that autistic children were less sensitive to advice. Error bars show 95% confidence intervals. Neurotypical RTs show data from 'peer-advised' children, matched to the autistic groups.

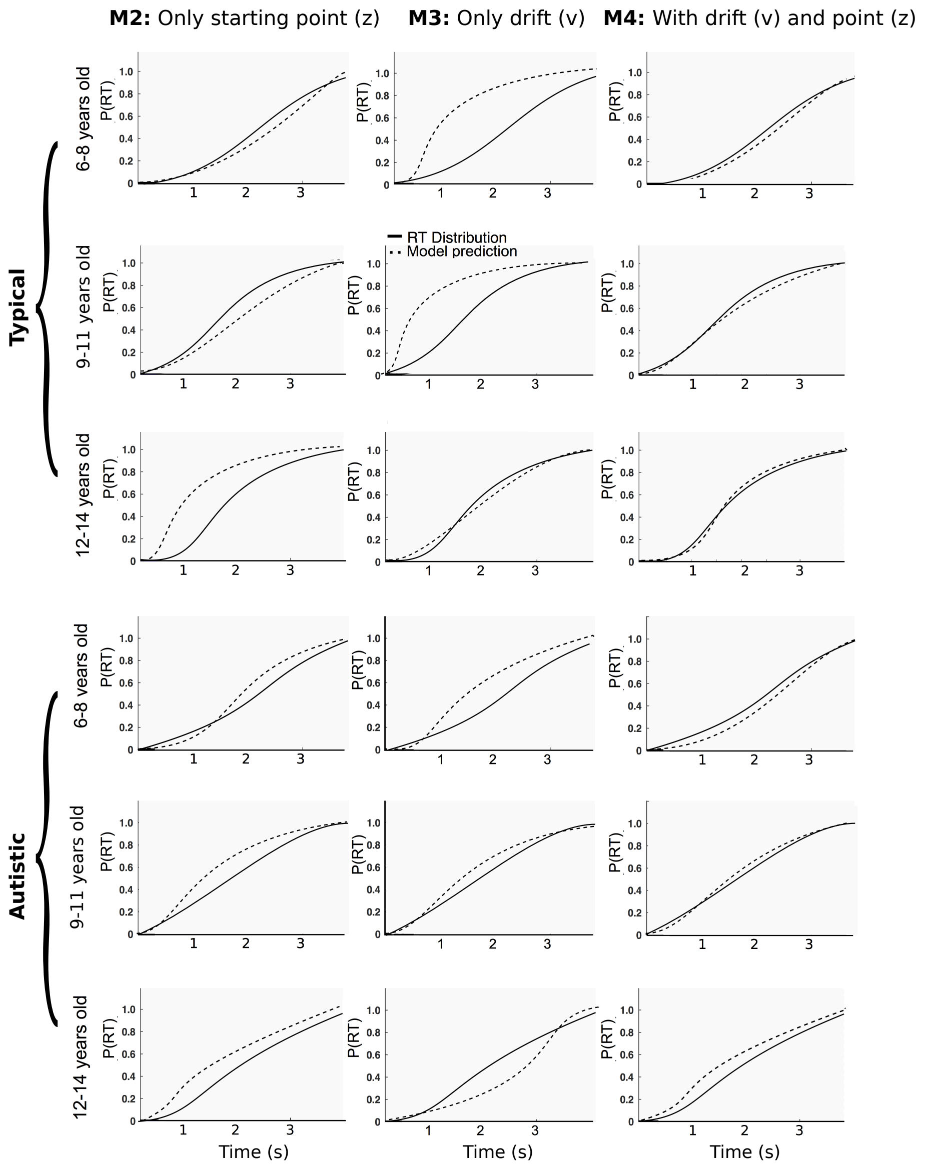

Fig. S3. Predicted and actual reaction time (RT) distributions. To assess goodness-of-fit, we compared the probability density functions of the actual RT distributions (solid line) with the RT distributions predicted with three main drift diffusion models we investigated (M2, M3, M4) (dashed line). Each graph plots the cumulative value of the chi-squared distribution against RT in seconds. The left column shows distributions for M2 which allowed only starting point to vary with social influence. The middle column shows M3 which only allowed drift to vary with social influence. The right column shows M4 which allowed both starting point and drift rate to vary with social influence. For all age and experimental groups, M4 provides a good match to the RT distribution, providing a significantly better match than M2 (chi-square, $p < 0.05$). Qualitatively, neurotypical 12-14 year-old children appear also well matched by M3 in contrast to the other ages and conditions, underlining that allowing drift rate to vary with social influence is important for the model fit in this group.

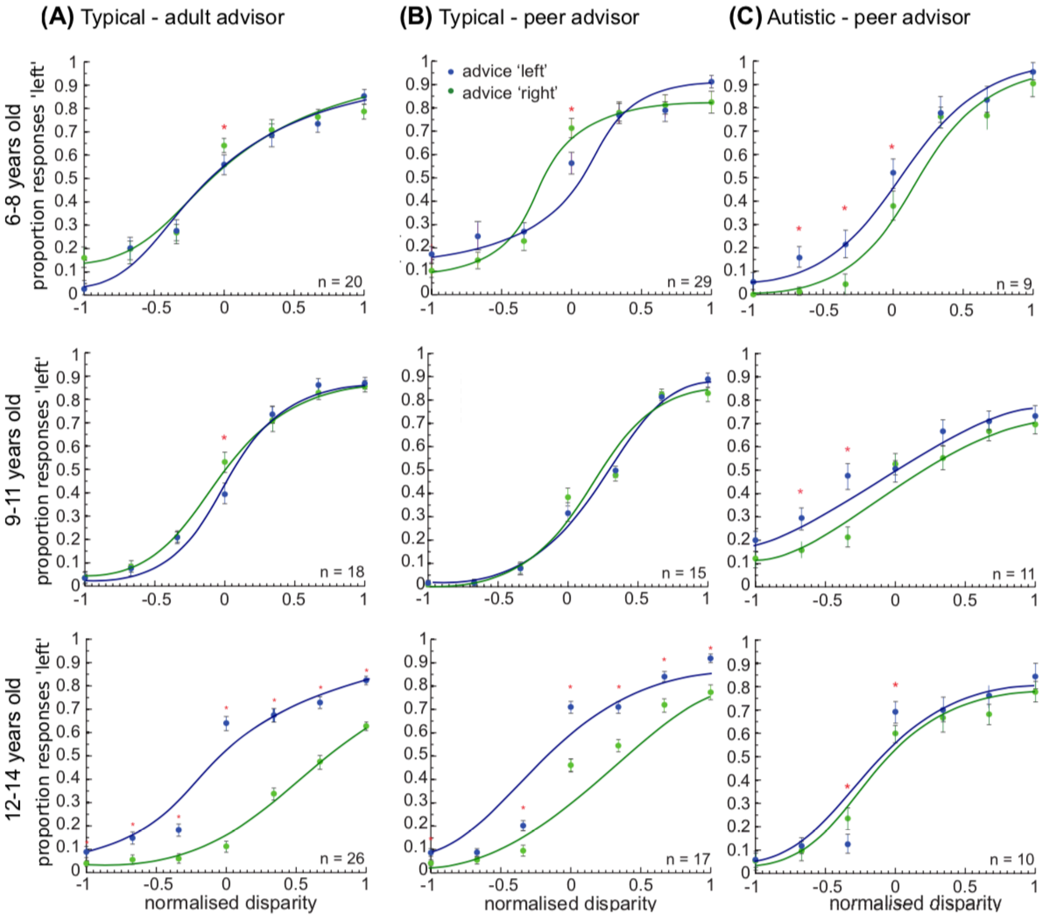

Fig. S4. Fitting behavioural responses with the drift diffusion model. To assess how well the drift diffusion model could predict behavioural responses in our study, we fitted the psychometric data from Figure 3 with drift diffusion Model M4. M4 allows both drift rate and starting point to vary with social influence direction. Conventions as in Figure 3. **(A)** Behavioural responses for neurotypical children under social influence of a gender-matched adult. Average behavioural responses were fitted with M4 separately for advice 'left' (blue line) and advice 'right' (green line). Error bars indicate standard error of the mean (SEM). * indicates a significant difference between responses for 'leftwards' versus 'rightwards' advice (Wilcoxon Rank Sum, $p < 0.05$, Bonferroni corrected). **(B)** Psychometric functions for neurotypical children with an age and gender-matched peer advisor. Same procedures and conventions as in (A). **(C)** Psychometric functions for autistic children with an age- and gender-matched peer advisor. Children were matched to the neurotypical groups in (B). Same procedures and conventions as in (A) & (B).

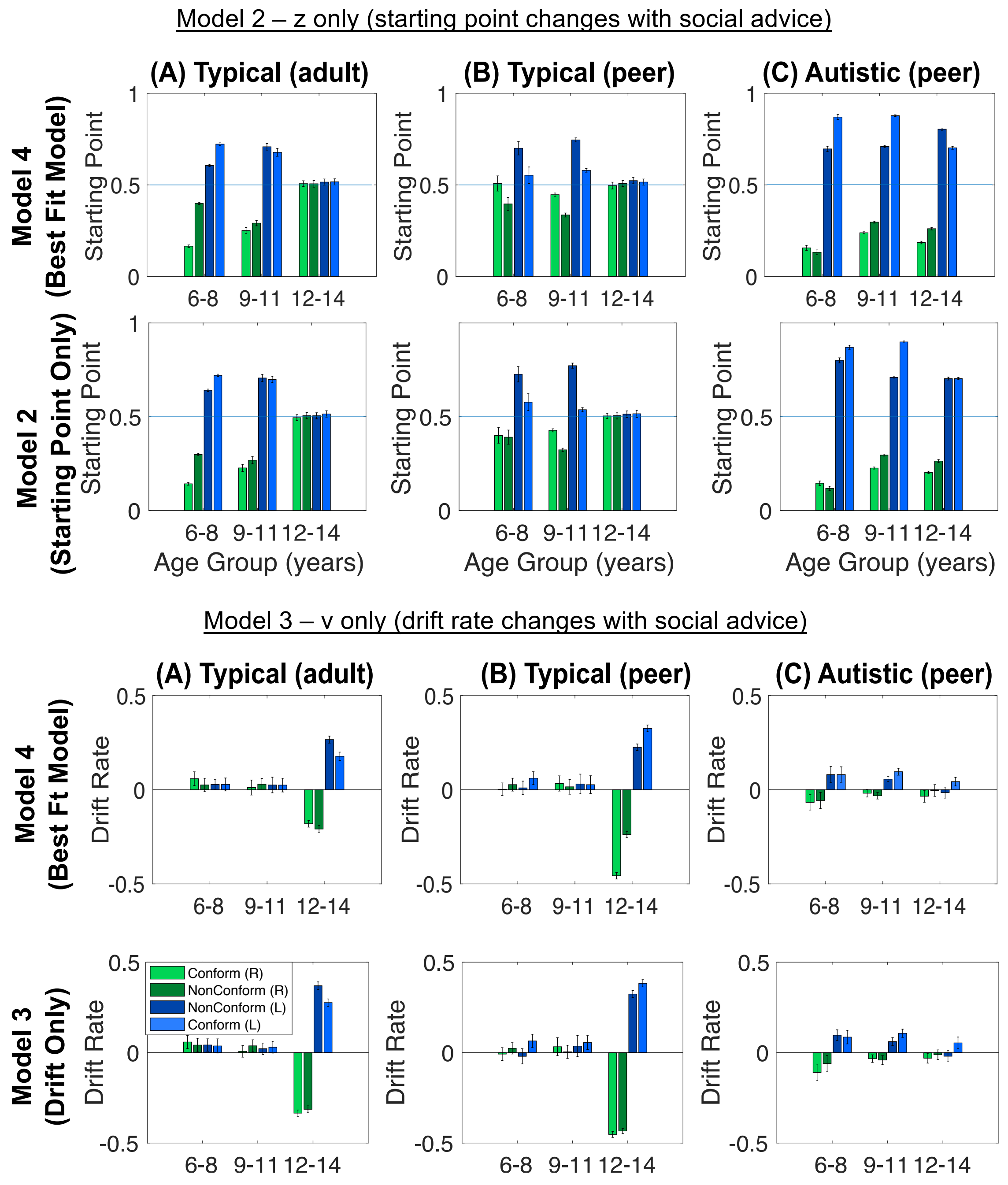

Fig. S5. Drift and Starting Point parameters for Model 2 (z only) and Model 3 (v only) with Model 4 (v and z). A comparison of starting point (z) and drift rate parameter (v) estimates between the models in which either starting point (Model 2) or drift rate (Model 3) alone were allowed to vary with social influence and the best-fitting Model 4, for which both parameters were allowed to vary with social influence.

1. Your co-pilot can not see which way the black holes are spinning.
2. You need to help out your co-pilot by telling them which way the black holes are spinning!
3. First, focus on the white square in the middle of the screen.
4. Tell your co-pilot when you are ready to start.
5. Keep looking at the white square! A black hole is about to appear.
6. When the black hole is gone, touch the screen to show which way you saw it spinning.
7. You can press 'LEFT' or 'RIGHT'.
8. Stay on your toes! When you have pressed which way the black hole is spinning, another will appear!

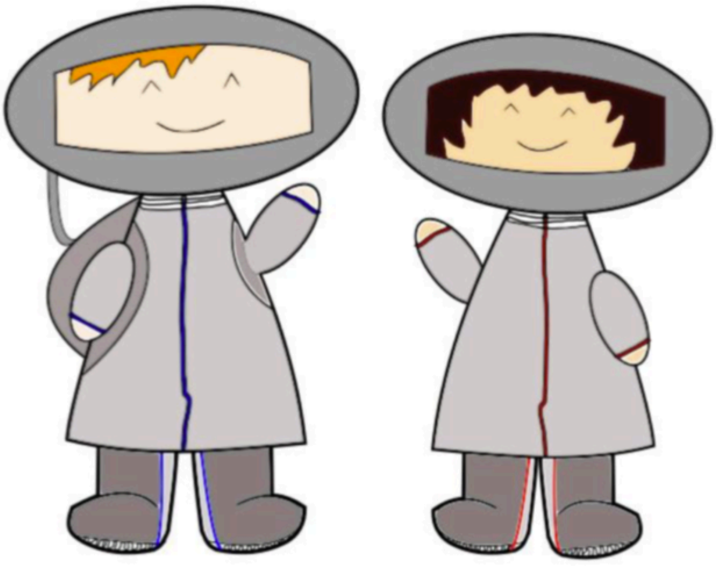

Fig. S6. Instructions provided to participants for the initial version of the task without social influence.

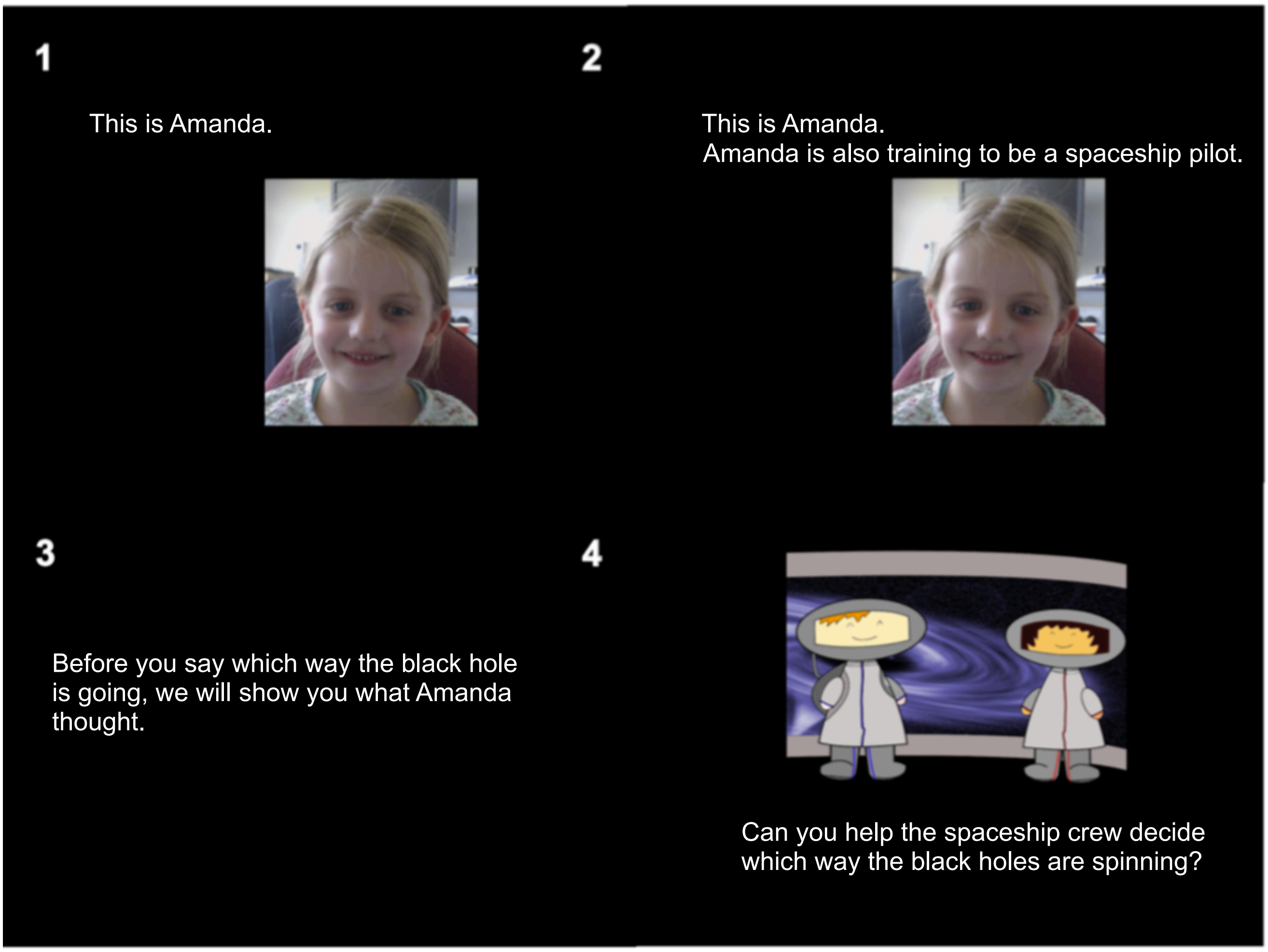

Fig. S7: Instructions provided to participants for the social influence task, introducing the social advisor.

Supplementary Tables

Neurotypical children - adult advisor											
**Age**	**Advice**			**Disparity**			**Gender**			**Age**	
years	F	p	df	F	P	df	F	p	df	F	p
6 to 8	2.424	0.119	1	140	<0.001*	6	2.797	0.079	1	143	<0.001*
9 to 11	3.357	0.067	1	112.432	<0.001*	6	2.006	0.157	1		
12 to 14	641.14	<0.001*	1	93.235	<0.001*	6	0.871	0.221	1		
**Neurotypical children - peer advisor**											
**Age**	**Advice**			**Disparity**			**Gender**			**Age**	
years	F	p	df	F	P	df	F	p	df	F	p
6 to 8	9.61	0.002*	1	496.41	<0.001*	6	0.992	0.2	1	119.9	<0.001*
9 to 11	0.805	0.37	1	734.003	<0.001*	6	1.245	0.192	1		
12 to 14	129.53	<0.001*	1	90.838	<0.001*	6	1.456	0.277	1		

Neurotypical children – overall													
**Advice**			**Advisor**			**Disparity**			**Gender**			**Age**	
F	p	df	F	p	df	F	p	df	F	p	df	F	p
100.48	<0.001*	1	0.046	0.8	1	152.24	<0.001*	6	1.53	0.22	1	163.4	<0.001*

Table S1. N-way ANOVA for effect of advice direction, disparity, gender, and age group on perceptual decisions of neurotypically developing children. Sensory evidence (binocular disparity) and age strongly affected judgements about the direction of cylinder rotation showing that the children could do the visual task. Peer social influence (advice) also had a significant effect on 6-8 year-old children, but not 9-11 year-olds nor with an adult advisor for the same age groups. For the 12-14 year-old children, we found a large, significant effect on behavioural responses in the direction of social influence. There was no significant effect of gender. * indicates a significant p-value (Bonferroni-corrected p < 0.05).

Typical<br>Age range	(Adult advisor)<br>n	Mean	std
(years)			
6.1-8.7	20	0.926	0.024
9.1-11.3	18	0.966	0.018
12.2-14.3	26	0.915	0.066
Average		0.945	0.032
**Typical<br>Age range**	**(Peer advisor)<br>n**	**Mean**	**std**
(years)			
6.5-8.6	29	0.938	0.023
9.2-11.7	15	0.970	0.030
12.1-14.5	17	0.934	0.037
Average		0.938	0.031
**Autistic<br>Age range**	**(Peer advisor)<br>n**	**Mean**	**STD**
**(years)**			
7.2-8.9	9	0.963	0.010
9.1-11.3	11	0.903	0.033
12.6-14.4	10	0.890	0.036
Average		0.916	0.042

Table S2. Mean adjusted R-squared (RSQ) values and standard deviations for the cumulative Gaussian fits of individual psychometric functions to perceptual choice data. RSQ value were high and comparable across all experimental groups – indicating generally that data was well fitted. Average values and standard deviations (SD) are shown split by social influence type, diagnosis and age group.

Autistic children – peer advice												
**Age**	**Advice**			**Disparity**			**Gender**			**Age**		
(years)	F	p	df	F	p	df	F	p	df	F	p	df
6 to 8	30.97	0.001*	1	492.347	<0.001*	6	0.311	0.602	1	4.14	0.016*	2
9 to 11	28.63	0.001*	1	133.282	<0.001*	6	0.502	0.478	1			
12 to 14	21.41	0.001*	1	207.61	<0.001*	6	0.756	0.388	1			

Table S3. N-way ANOVA for effect of advice direction, disparity, gender and age group on perceptual decisions of autistic children. For the autistic children, social influence (advice) had a significant effect on the choices of all age groups. An asterisk indicates a significant p- value (Bonferroni-corrected $p < 0.05$). There was no significant effect of gender.

Typical	(Adult)			
**Age range**	**n**	**WASI-II**	**SCQ**	
(years)		mean ±SD (range)	mean ±SD (range)	
6.1-8.7	20	102.3±5.2 (94-111) (n=11)	2.7±1.3 (0-6) (n=11)	
9.1-11.3	18	109.5±7.8 (89-124) (n=11)	2.1±1.1 (1-3) (n=11)	
12.2-14.3	26	104.3±5.1 (96-110) (n=18)	2.4±2.0 (1-5) (n=18)	
**Typical**	**(Peer)**			
**Age range**	**n**	**WASI-II**	**SCQ**	
(years)		mean ±SD (range)	mean ±SD (range)	
6.5-8.6	29	104.1±4.2 (92-108)	3.2±2.1 (0-12)	
9.2-11.7	15	108.3±6.7 (95-112)	2.5±1.8 (2-3)	
12.1-14.5	17	105.8±6.4 (94-121)	2.8±2.3 (1-6)	
**Autistic**	**(Peer)**			
**Age range**	**n**	**WASI-II**	**SCQ**	**ADOS-2**
**(years)**		mean ±SD (range)	mean ±SD (range)	mean ±SD (range)
7.2-8.9	9	103.4±9.8 (95-112)	21.5±4.3 (15-23)	12.3 ±2.5 (8-17)
9.1-11.3	11	109.5±10.1 (94-122)	26.1±3.8 (16-38)	16.7±3.9 (8-27)
12.6-14.4	10	102.3±8.6 (92-113)	24.2±5.1 (15-31)	10.9±2,9 (9-12)

Table S4. Descriptive statistics for the two neurotypical experimental groups and the autistic group of children, as a function of age group. WASI-II (Wechsler Abbreviated Scales of Intelligence, 2nd edition, Wechsler, 2011) reports IQ scores (population M = 100; SD = 15). SCQ is the Social Communication Questionnaire (Rutter et al., 2003) and ADOS-2 is the Autism Diagnostic Observation Schedule (Lord et al., 2012). For both the SCQ and the ADOS-2, higher scores reflect greater degrees of autistic symptoms.

	Age	*a*	T_{er}	*eta*	*sz*	*st*
**Typical (adult)**	6 to 8	0.6766	0.3934	0.0001	0.03087	0.74124
	9 to 11	0.6075	0.631	0.00886	0.04392	0.46107
	12 to 14	0.5428	0.3222	0.0002	0.02476	0.62838
**Typical (peer)**	6 to 8	0.6779	0.3181	0.00321	0.05522	0.6351
	9 to 11	0.5995	0.4371	0.01558	0.01044	0.33019
	12 to 14	0.6383	0.4568	0.01902	0.04891	0.55453
**Autistic**	6 to 8	0.6443	0.315	0.002	0.0431	0.6541
	9 to 11	0.6566	0.4651	0.0042	0.04456	0.7194
	12 to 14	0.5634	0.6342	0.00024	0.05109	0.6491

Table S5. Values for other parameters in the best fitting drift diffusion model (Model 4: drift rate and starting point may vary with social influence). The parameters shown here were allowed to vary with disparity, but not with social influence condition: ***a*** - boundary separation, $\mathbf{T}_{er}$ - non-decision time, ***eta*** - intertrial standard deviation of drift rate, ***sz*** - the range of starting point values and ***st*** - the range of non-decision time.
